# The effect of RNA base lesions on mRNA translation

**DOI:** 10.1093/nar/gkv377

**Published:** 2015-04-20

**Authors:** Alessandro Calabretta, Pascal A. Küpfer, Christian J. Leumann

**Affiliations:** Department of Chemistry and Biochemistry, University of Bern, Freiestrasse 3, 3012 Bern, Switzerland

## Abstract

The biological effect of oxidatively damaged RNA, unlike oxidatively damaged DNA, has rarely been investigated, although it poses a threat to any living cell. Here we report on the effect of the commonly known RNA base-lesions 8-oxo-rG, 8-oxo-rA, ε-rC, ε-rA, 5-HO-rC, 5-HO-rU and the RNA abasic site (rAS) on ribosomal translation. To this end we have developed an *in vitro* translation assay based on the mRNA display methodology. A short synthetic mRNA construct containing the base lesion in a predefined position of the open reading frame was ^32^P-labeled at the 5′-end and equipped with a puromycin unit at the 3′-end. Upon *in vitro* translation in rabbit reticulocyte lysates, the encoded peptide chain is transferred to the puromycin unit and the products analyzed by gel electrophoresis. Alternatively, the unlabeled mRNA construct was used and incubated with ^35^S-methionine to prove peptide elongation of the message. We find that all base-lesions interfere substantially with ribosomal translation. We identified two classes, the first containing modifications at the base coding edge (ε-rC, ε-rA and rAS) which completely abolish peptide synthesis at the site of modification, and the second consisting of 8-oxo-rG, 8-oxo-rA, 5-HO-rC and 5-HO-rU that significantly retard full-length peptide synthesis, leading to some abortive peptides at the site of modification.

## INTRODUCTION

The integrity of nucleic acids is constantly challenged by ubiquitous reactive oxygen and nitrogen species (ROS, RNS) which are mainly produced endogenously by the mitochondrial respiratory chain (NADPH oxidases (NOX), and 5-lipoxygenases (LOX)), or originate from exogenous sources like tobacco, drugs, xenobiotics and ionizing radiation (1). These agents primarily oxidize the nucleic acid bases and are thus a threat for the genetic information transfer. While distinct repair mechanisms are present to maintain the integrity of the genome, the fate of oxidatively damaged RNA is still largely unknown: so far, only in the case of methylated RNA a repair mechanism could be identified (2). Recently, high levels of oxidatively damaged RNA have been closely linked to aging and to a still growing number of neurodegenerative and other diseases (3). The data so far suggest that RNA oxidation is an early event in these processes, rather than merely the effect of cell decay (4,5).

Nucleobases can be either directly oxidized by ROS or indirectly by oxidation products from polyunsaturated fatty acids (PUFAs). For direct oxidation of nucleic acids the main reactive oxygen species are singlet oxygen (^1^O_2_) and the hydroxyl radical (•OH) that can yield a large number of compounds in DNA (6,7) and presumably also in RNA. Oxidized RNA nucleosides isolated so far include 5-hydroxyuridine (5-HO-rU), 5-hydroxycytidine (5-HO-rC), 8-oxo-7,8-dihydroguanosine (8-oxo-rG) and 8-oxo-7,8-dihydroadenosine (8-oxo-rA) (8,9). In parallel to the formation of 8-oxo purines, intermediate 8-hydroxy-7,8-dihydro-7-yl radicals that form by the abstraction of hydroxyl radicals on C8 can also undergo single electron reduction to form 2,6-diamino-4-hydroxy-5-formamidopyrimidine (FapyG) and 4,6-diamino-5-formamidopyrimidine nucleosides (FapyA), respectively (10). Both lesions have recently been quantified in the analysis of RNA damage in Alzheimer's disease patients. It can therefore be assumed that they are also present in the pool of oxidatively damaged RNA (11). In addition, Akaike *et al*. showed that nitric oxide and peroxynitrite accelerate mutation of RNA viruses *in vitro* as well as *in vivo* (12). Presumably, this effect is based on the mutagenic properties of 8-oxo-rG and 8-nitro-rG that are formed under these RNS conditions (13). Nucleobases can also be alkylated by lipid peroxidation products: PUFAs are oxidized enzymatically (lipoxygenases, cyclooxygenases) or by direct abstraction of hydroxyl radicals to a complex mixture of hydroperoxides (14). Subsequent products have been shown to yield a great number of adducts with nucleobases in DNA (15). In particular 4,5-epoxy-2-decanal (EDE) and 4-hydroperoxy-2-nonenal (HPNE) have been shown to yield unsubstituted etheno adducts with dA, dG and dC.

**Figure 1.**
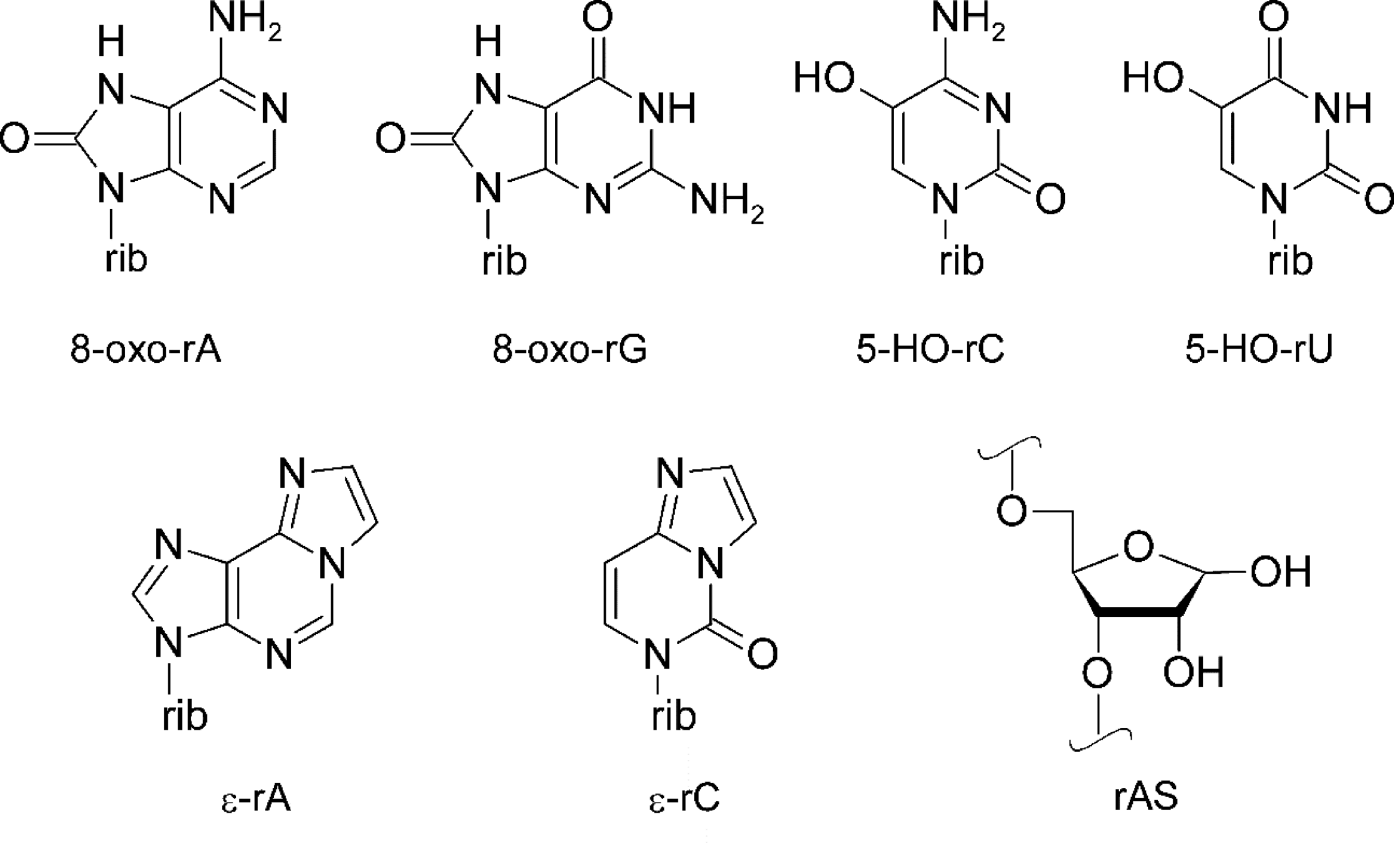
Structures of RNA lesions investigated: 8-oxo-7,8-dihydroadenosine (8-oxo-rA), 8-oxo-7,8-dihydroguanosine (8-oxo-rG), 5-hydroxycytidine (5-HO-rC), 5-hydroxyuridine (5-HO-rU), 1,*N*^6^-ethenoadenosine (ε-rA), 3,*N*^4^-ethenocytidine (ε-rC) and abasic site (rAS); rib = ribose.

In order to elucidate their potential mutagenic properties, various oxidatively damaged nucleobases have been synthetically incorporated into RNA and their coding preferences in templated primer extension reactions (PEX) by reverse transcriptases have been examined. These studies include base lesions such as abasic sites (rAS) (16,17), 8-oxo-rA (18), 8-oxo-rG (19), 5-HO-rU (20), 5-HO-rC (21), ε-rA and ε-rC (22) (Figure 1). The findings suggest that some of these oxidized bases change their native Watson–Crick base-pairing selectivity. In the context of mRNAs this might subsequently lead to the synthesis of modified proteins, as was suggested earlier (23).

The possible impact of oxidative lesions on the translation machinery has only been addressed by a handful of studies so far. Translation of oxidized firefly luciferase mRNA (treated with H_2_O_2_/cytochrome c) in rabbit reticulocyte lysate and transfected HEK-293 and PC-12 cells showed that luciferase activity and detectable protein levels decreased dramatically in all systems depending on the oxidation level of the mRNA (24). Similar results were obtained for the translation of oxidized enhanced green fluorescent protein mRNA in transfected HEK-293 cells. Ribosome stalling on the transcript rather than the production of truncated proteins (4) was observed in these cases. In another study Tanaka *et al*. showed that translation of luciferase mRNA in reticulocyte lysate and in HEK-293 cells led to increased levels of short polypeptides, independent whether the mRNA was oxidized before translation (treated with Fe/ascorbate/H_2_O_2_) or translation was performed under oxidative conditions (in the presence of paraquat) (25). The results indicate that the stability of oxidized mRNA is comparable to that of non-oxidized mRNA and that oxidized mRNA is in principal accepted for translation.

To understand the impact of mRNA base-lesions on a molecular level we set out to study the translation of short synthetic mRNAs with a series of well-defined single oxidative lesions in predefined positions using the *in vitro* RNA display system of Roberts and Szostak (26). In this system, the synthesized peptide is covalently linked to the encoding mRNA bearing a puromycin unit on the 3′-end of the template. This system allows the investigation of the effect of single oxidative lesions on translation and the direct analysis of the fusion products by gel electrophoresis.

## MATERIALS AND METHODS

### Oligonucleotide synthesis and purification

Unmodified DNA and RNA oligonucleotides were purchased from Microsynth (Balgach, Switzerland) and HPLC- or PAGE-purified where necessary. Modified oligoribonucleotides were synthesized using the known 2′-O-TBDMS protected phosphoramidite building blocks for the base-oxidized nucleosides of Figure 1 (18,20,22,27,28). In the case of 8-oxo-rG the synthesis of the phosphoramidite building block (19) was slightly changed (see the Supplementary Information). Natural 2′-O-TBDMS protected RNA phosphoramidites and CPG solid supports were from GlenResearch or AZCO. Puromycin solid support and spacer phosphoramidite 9 were purchased from GlenResearch. Syntheses were performed on a 1.3-μmole scale on a Pharmacia Gene Assembler Plus DNA synthesizer following standard phosphoramidite protocols with 5-(ethylthio)-1*H*-tetrazole (0.25 M in acetonitrile) as the activator and coupling times of 12 min. Table 1 gives an overview over all oligoribonucleotides used in this study.

**Table 1.** DNA and RNA sequences used in this work and cleavage conditions

splint	d(TTTTTTTTTTGATCAGTTTCTGTTC)	a)
linker	d(AAA AAA AAA AAA AAA AAA AAA-999-ACC-P)	NH_4_OH/EtOH 3:1, 55°C, 24 h
RNA1	r(GGG AGG ACG AAA UGG AAC AGA **A**AC UGA UC)	a)
RNA2	r(GGG AGG ACG AAA UGG AAC AGA **C**AC UGA UC)	a)
RNA3	r(GGG AGG ACG AAA UGG AAC AGA **G**AC UGA UC)	a)
RNA4	r(GGG AGG ACG AAA UGG AAC AGA **U**AC UGA UC)	a)
RNA5	r(GGG AGG ACG AAA UGG AAC AGA **(ε-rA)**AC UGA UC)	NH_4_OH/EtOH 3:1, rt, 17 h
RNA6	r(GGG AGG ACG AAA UGG AAC AGA **(5-HO-rU)**AC UGA UC)	NH_4_OH/EtOH 3:1, rt, 17 h
RNA7	r(GGG AGG ACG AAA UGG AAC AGA **(8-oxo-G)**AC UGA UC)	NH_4_OH/EtOH 3:1, 0.25-M ethanethiol, 55°C, 17 h
RNA8	r(GGG AGG ACG AAA UGG AAC AGA **(5-HO-rC)**AC UGA UC)	NH_4_OH/EtOH 3:1, 55°C, 17 h
RNA9	r(GGG AGG ACG AAA UGG AAC AGA **(8-oxo-A)**AC UGA UC)	MeNH_2_ (40% in H_2_O)/MeNH_2_ (33% in EtOH) 1:1, rt, 17 h
RNA10	r(GGG AGG ACG AAA UGG AAC AGA **(ε-rC)**AC UGA UC)	NH_4_OH/EtOH 3:1, rt, 17 h
RNA11	r(GGG AGG ACG AAA UGG AAC AGA **(rAS)**AC UGA UC)	NH_4_OH/EtOH 3:1, 55°C, 17 h

a) Purchased from Microsynth, Balgach, Switzerland. P = puromycin, 9 = triethylene glycol spacer. Bold letters refer to the position of the modifications.

Modified oligoribonucleotides were deprotected using the conditions reported in Table 1. Solid supports were filtered off and washed with water and solutions were evaporated to dryness at room temperature in a Speedvac evaporator. The resulting pellets were further dried by addition and evaporation of dry ethanol. The obtained product was dissolved in anhydrous DMSO (100 μl) and neat triethylamine trihydrofluoride (125 μl) was added. After shaking at 25°C for 24 h, an aqueous solution of NaOAc (3 M, pH = 5.2, 25 μl) and n-butanol (1 ml) were added and the mixture was chilled on dry ice for 45 min. After centrifugation for 20 min in an Eppendorf centrifuge the supernatant was discarded, the pellet washed twice with cold ethanol (80%, 1 ml) and dried under high vacuum at room temperature, yielding the crude oligonucleotides.

Oligoribonucleotides were purified on a preparative 20% denaturing polyacrylamide gel, product bands electroeluted with an Elutrap electroelution system (Schleicher & Schuell) and desalted using Amicon ultra 0.5 filters (MWCO 3 KDa, Millipore). Oligonucleotide concentrations were determined using a NanoDrop ND-100 UV/Vis spectrophotometer (NanoDrop Technologies, Inc.). Stock solutions were made from DEPC treated H_2_O. Oligoribonucleotides containing (2-nitrophenyl)ethyl or 2-nitrobenzyl protections (rAS and 5-HO-rC) were deprotected with UV-light at wavelengths above 300 nm just before the translation assays (see below).

### Splint ligation

In order to create the translation templates, each RNA sequence 1–11 was fused to the 5′-phosphorylated end of the linker sequence (29) using the splint oligonucleotide and T4 DNA ligase (30). Forty two microliter of linker sequence (100 μM) was mixed with 28 μl of 10X ligase buffer (300-mM Tris-HCl, 100-mM MgCl_2_, 100-mM DTT and 10-mM adenosine triphosphate (ATP), pH 7.8), 140 μl of water and 42 μl of T4 polynucleotide kinase (Fermentas). The mixture was incubated at 30°C for 30 min, 75°C for 10 min and 20°C for 10 min. Sixty three microliter of the splint (100 μM) and 42 μl of an RNA sequence (100 μM) were added, and the solution was incubated at 80°C for 2 min and 30°C for 20 min. Fourteen microliter of 10X ligase buffer, 14 μl of T4 DNA ligase and 40 μl of PEG were added, and the mixture was incubated at 30°C for 3 h and 70°C for 5 min.

The ligation products were purified on preparative 20% denaturing polyacrylamide gels, electroeluted with an Elutrap electroelution system (Schleicher & Schuell) and desalted using Amicon ultra 0.5 filters (MWCO 3 KDa, Millipore). The translation templates were precipitated (100 μl of water, 30 μl of 3-M NaOAc and 1.2 ml of ethanol), washed with 80% ethanol, dried and diluted in water. Oligonucleotide concentrations were determined using a NanoDrop ND-100 UV/Vis spectrophotometer (NanoDrop Technologies, Inc.).

### Labeling of the synthetic mRNAs

The splint ligation product was phosphorylated with [γ-^32^P]ATP (Hartmann Analytic) and T4 polynucleotide kinase (Fermentas) as follows: template (30 pmol), [γ-^32^P]-ATP (60 mCi) and T4 PNK (10 U) were incubated in a total volume of 20 μl at 37°C for 30 min in T4 PNK buffer (50-mM Tris-HCl, 10-mM MgCl_2_, 5-mM DTT, 0.1-mM spermidine, 0.1-mM ethylenediaminetetraacetic acid (EDTA), pH 7.6). T4 PNK was inactivated by heating the sample to 80°C for 2 min. The labeled oligonucleotide was purified on a preparative 20% denaturing polyacrylamide minigel. Product bands were cut, crushed and suspended in elution buffer (0.5-M Tris-HCl, 0.1% sodium dodecyl sulphate, 0.1-mM Na_2_EDTA, 1-mM MgCl_2_, pH 7). To each crushed band 1 ml of elution buffer was added and the mixtures were frozen and melted to room temperature three times. The mixtures were incubated overnight at room temperature. The supernatant was recovered after centrifugation and the solid was washed with elution buffer. Solutions containing oligonucleotides were concentrated with n-butanol and precipitated with ethanol and 3-M NaOAc. Oligonucleotides were dissolved in DEPC treated water and used directly for translation assays.

### Translation assays

Translation assays were performed following the protocol developed by Roberts and Szostak (26) using commercially available rabbit reticulocyte lysates (Flexi® Rabbit Reticulocyte Lysate System, Promega). Before translation, the RNA template was denatured for 3 min at 65°C and left for 20 min on ice. In the case of abasic site or 5-HO-rC containing oligonucleotides, the photocleavable protecting groups were removed just before the assay by irradiation for 1 h using a slide projector (17,21). The translation mastermix was prepared using stock solutions provided with the lysate as follows: 33 μl of lysate, 1 μl of 1-mM amino acid mixture, 0.5 μl of 25-mM Mg(AcO)_2_, 1 μl of 2.5-M KCl, 1 μl of 100-mM DTT, 1 μl of RNasin (Promega), RNA template (20 pmol in 12 μl) and 0.5 μl of water. When [^35^S]-methionine labeling was used, an amino acid mixture without methionine was used, and 2 μl of [^35^S]-methionine (1200 Ci/mmol at 10 mCi/ml, Promega) was added. The system was incubated for 90 min at 37°C and for 40 min at 0°C. Then, 8 μl of 4-M KCl and 5 μl of 1-M MgCl_2_ were added and the system was incubated for 1 h at room temperature and left overnight at −20°C. The mixture was diluted in gel loading buffer (98% formamide, 0.05% xylene cyanol (FF), 0.05% bromophenol blue), heated to 70°C for 5 min and applied to a 10 or 20% denaturing polyacrylamide gel. Radioactivity was detected and quantified on a Storm 820 phosphorimager with ImageQuant software (GE Healthcare).

## RESULTS

### Experimental setup

**Figure 2.**
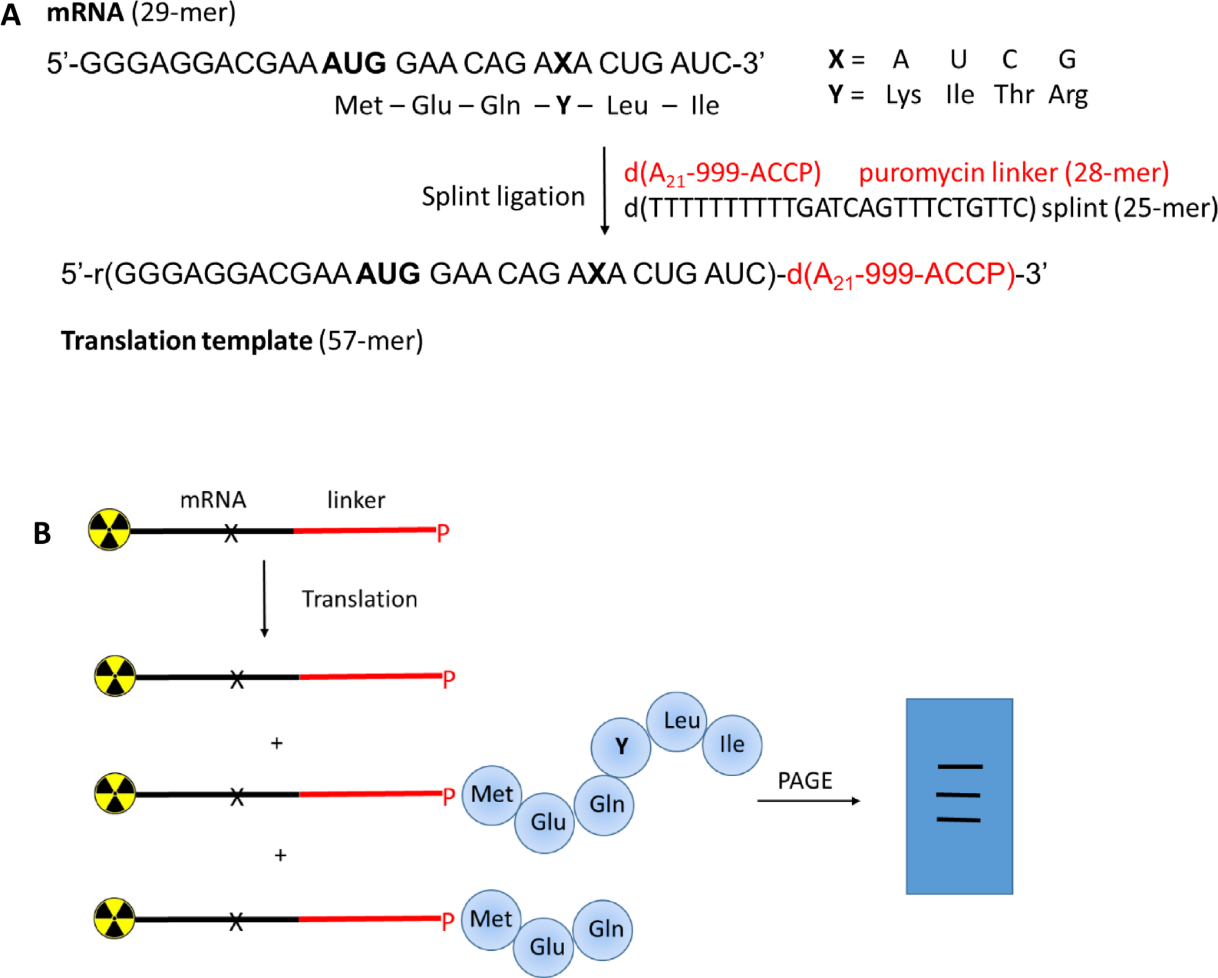
(**A**) Synthesis of the puromycin mRNA constructs via splint ligation of synthetic oligonucleotides. X is the position where the lesions were introduced and Y indicates the encoded amino acids, 9 = triethylene glycol linker, P = puromycin. (**B**) Translation assay: the hybrid template is labeled with a radioactive phosphate and used in a standard rabbit reticulocyte translation assay. The results are analyzed by PAGE.

To determine the effect of an mRNA lesion on protein biosynthesis we decided to develop an *in vitro* translation assay (Figure 2) based on the mRNA-display method (26,29,31,32). In this method the encoded peptide becomes covalently linked to the message at the end of translation by a puromycin unit (P) that is attached via a DNA-linker to the 3′-end of the message. Puromycin is a nucleoside that mimics the aminoacyl end of tRNA and covalently binds the nascent peptide by entering the P-site of the ribosome (26). We reasoned that this method is superior to standard translation assays without covalent attachment of the peptide in view of expected difficulties in the isolation of the small peptide out of the translation mixture. Furthermore, the nucleic acid part in the translation product enables easy analysis by polyacrylamide gel electrophoresis (PAGE) from which information on full length, partial length or absence of an attached peptide should be visible by differential gel shifts. ^32^P-labeling of the mRNA construct before translation can be used to visualize the reaction products. Alternatively, translation with unlabeled mRNA in the presence ^35^S-methionine can be used to unambiguously prove the covalent inclusion of the peptide.

To this end we designed short synthetic mRNA 29 mers (Figure 2a) encoding 6 amino acids containing each of the base-modifications depicted in Figure 1 in a predefined position (X). These mRNAs were then fused to a DNA-linker consisting of 21 dA followed by three ethylene glycol units and the dACCP domain via splinted ligation, leading to the final transcription template.

### Sequence design and synthesis

The assay is illustrated in Figure 2b and corresponds to a modified sequence construct used in previous work (26). The mRNA was designed bearing a ribosome binding site at the 5′ end, followed by a start codon (AUG) and five more codons. The linker sequence consisted of 21 dA units, linked to three ethylene glycol spacers, a CAA sequence and a puromycin unit. Spacers were introduced in order to increase template flexibility and with this fusion efficiency (29). The lesions were placed into the second position of the fourth codon. Thus, read-through or abortion of peptide synthesis was expected to be easily detectable by a shift of the product band on an electrophoretic gel.

The synthesis of oligoribonucleotides containing the base lesions was performed using standard RNA protocols. To prevent degradation at the site of the base lesions, cleavage and deprotection conditions had to be optimized for each lesion individually (see the Materials and Methods section). Deprotected sequences were purified by PAGE, electroeluted and desalted before use. Oligoribonucleotides containing an abasic site or a 5-HO-rC unit, bearing a photocleavable protecting group, were additionally light deprotected directly before use.

Full-length mRNA constructs were obtained via splint ligation (Figure 2). The efficiency of the ligation was monitored by PAGE, and was estimated to be quantitative in all cases (an example of a gel is included in the Supplementary Information section). The mRNA-puromycin constructs were again purified by PAGE and labeled with [γ-^32^P] ATP. Fusion translation experiments were carried out as reported in the Materials and Methods section, using rabbit reticulocyte lysate. The experiment was performed in two phases, a translation phase followed by a peptide-mRNA fusion phase induced by increasing the salt concentration. Final mixtures were analyzed by 10% PAGE.

**Figure 3.**
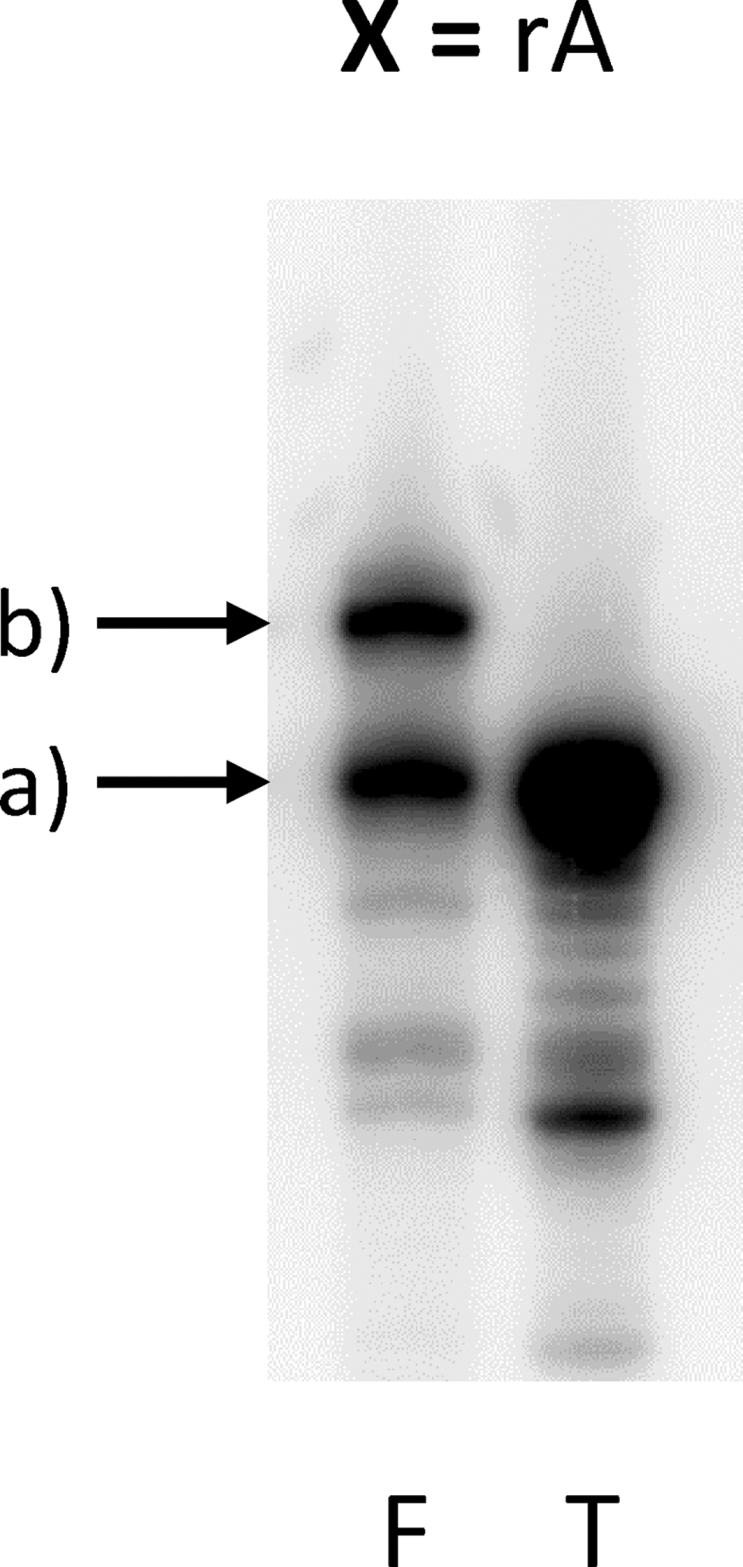
Fusion-translation product of an mRNA construct bearing a rA at position X (X = A). T = template; F = fusion-translation mixture; a) = template; b) = fusion product.

In order to study the efficiency of the translation assay, a template using a natural rA unit at position X was used. As shown in Figure 3, the product of the mRNA display assay shows two main bands. The faster eluting band (a) can be attributed to the non-translated template while the slower moving band (b) was attributed to the template-peptide fusion product.

**Figure 4.**
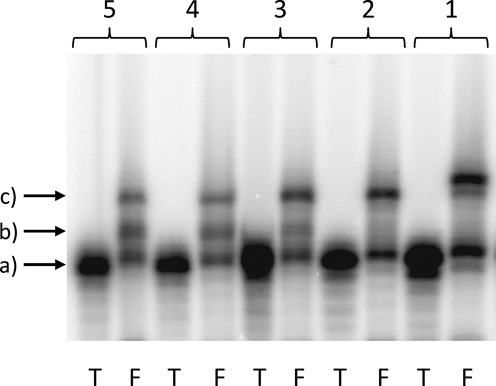
Fusion-translation product obtained from the mRNA construct containing X = rC or X = 5-HO-rC. Column 1: rC; column 2: 5-HO-rC (5-O-protected); columns 3–5: 5-HO-rC (5-O-deprotected) using differently prepared mRNA constructs; column 3: 5-O-deprotected before translation; column 4: 5-O-deprotected before splint ligation; column 5: 5-O-deprotected before the splint ligation without subsequent heating. Lane F: fusion-translation assay products; lane T: mRNA construct as control. Arrows: a) = template; b) = truncated fusion product; and c) = full-length fusion product.

We then tested the effect of the base lesion 5-HO-rC on translation. As reported in a previous paper, this lesion showed some unexpected thermal melting properties when introduced into an RNA sequence (27). An mRNA construct containing unmodified rC was used as a control. To identify whether different pretreatments of the mRNA construct had an effect on translation we performed the assay with 5-HO-rC that was base-deprotected either before or after splint ligation and with and without heat treatment. Since the 5-O-protected 5-HO-rC construct was anyway available it was also tested. The results of the translation assay are depicted in Figure 4.

From entries 3–5 (Figure 4) it appears that translation of a 5-HO-rC unit as compared to an rC unit (entry 1) leads to an overall reduced translation efficiency. More importantly, intermediates appear that correspond to fusion products with aborted peptide sequences most likely at the site of lesion. From comparison of entries 3–5 it becomes clear that the appearance of truncated transcripts is independent of the pretreatment of the mRNA construct before translation. We see, however, differences in translation efficiencies. The highest yields are obtained if the 5-HO-rC is deprotected just before the translation assay. Interestingly, if 5-O-protected 5-HO-rC was used (Figure 4, column 2), translation proceeded to the fusion product without apparent formation of intermediates. Obviously the introduction of a bulky benzyl group at O5 does not significantly interfere with translation.

**Figure 5.**
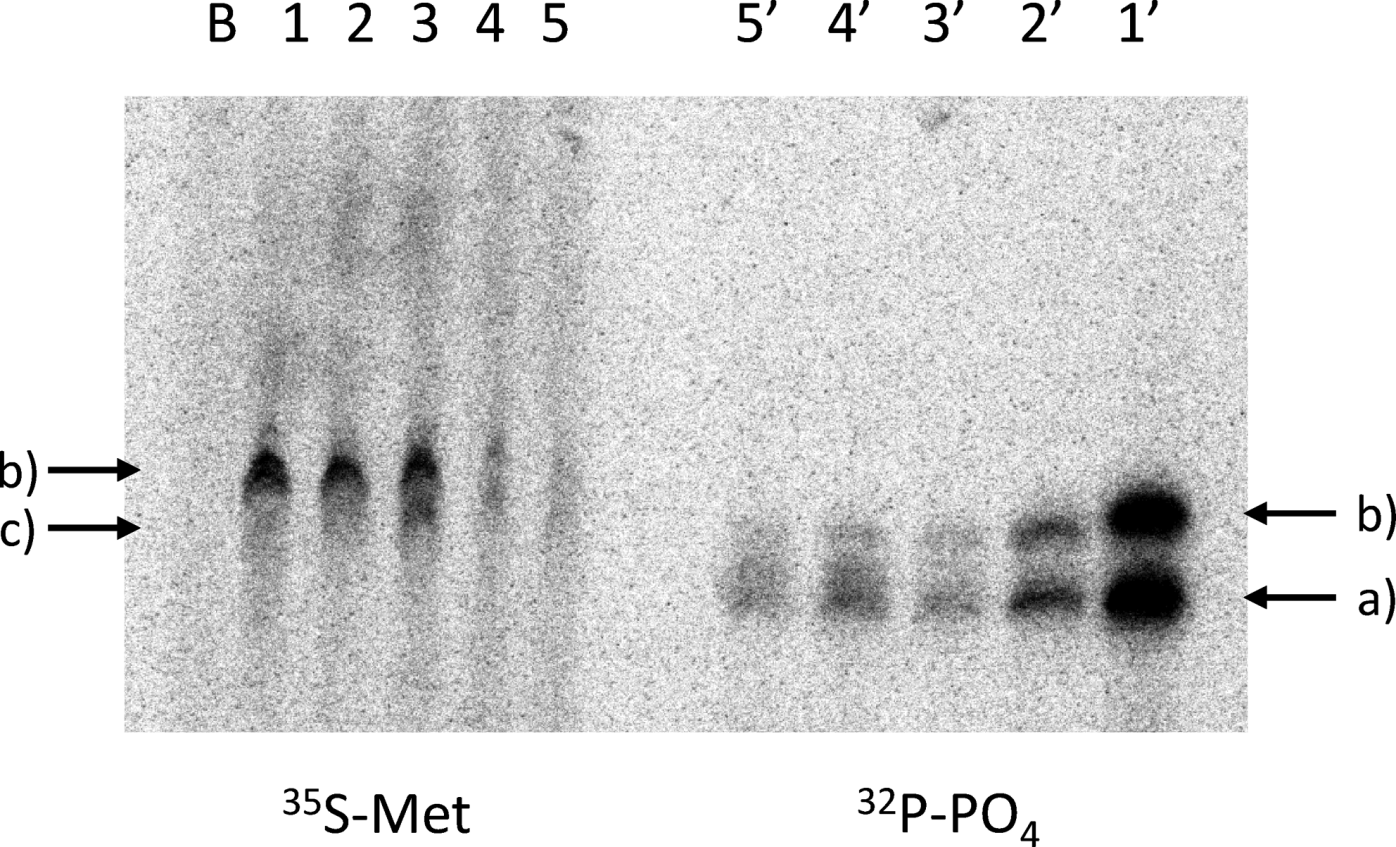
Fusion-translation experiment of Figure 4 using ^35^S-Met and non-labeled mRNA construct (left, lanes 1–5) or ^32^P-labeled template and non-labeled Met (right, lanes 1′–5′). Samples 1–5 are as in Figure 4; B corresponds to a fusion-translation experiment with ^35^S-Met without mRNA construct. Arrows: a) = template; b) = fusion product; and c) = truncated fusion products.

To further confirm the presence of the peptide in the fusion-translation products, the same experiment was repeated with a non-labeled mRNA construct and ^35^S-Met in the translation mixture. The corresponding fusion translation product that was then compared with the same product using the ^32^P-labeled construct (Figure 5). The ^32^P-labeled products were repeatedly diluted until the bands showed comparable intensity in the gel. As expected in the absence of the mRNA construct, no band arising from the fusion product is visible despite the presence of ^35^S-methionine in the mixture (Figure 5, lane B). However, for the case of X = rC, and the O-5-protected 5-HO-rC clear bands are visible (lanes 1 and 2) that have the same electrophoretic mobility as the corresponding bands in lanes 1′ and 2′, arising from the ^32^P-labeled mRNA construct. When using the unprotected 5-HO-rC lesion (lane 3) two bands are detectable representing the full-length and the aborted fusion translation products. Lanes 4 and 5, finally, show only very faint bands, in agreement with a lower efficiency of translation. The corresponding experiments with the ^32^P-labeled mRNA construct gave comparable bands, including, of course, those of the non-translated mRNA construct that shows the highest mobility (lanes 3′–5′). Thus this experiment unambiguously confirms that mRNA–peptide conjugates are formed during *in vitro* translation and that truncation is associated with incomplete peptide synthesis.

**Figure 6.**
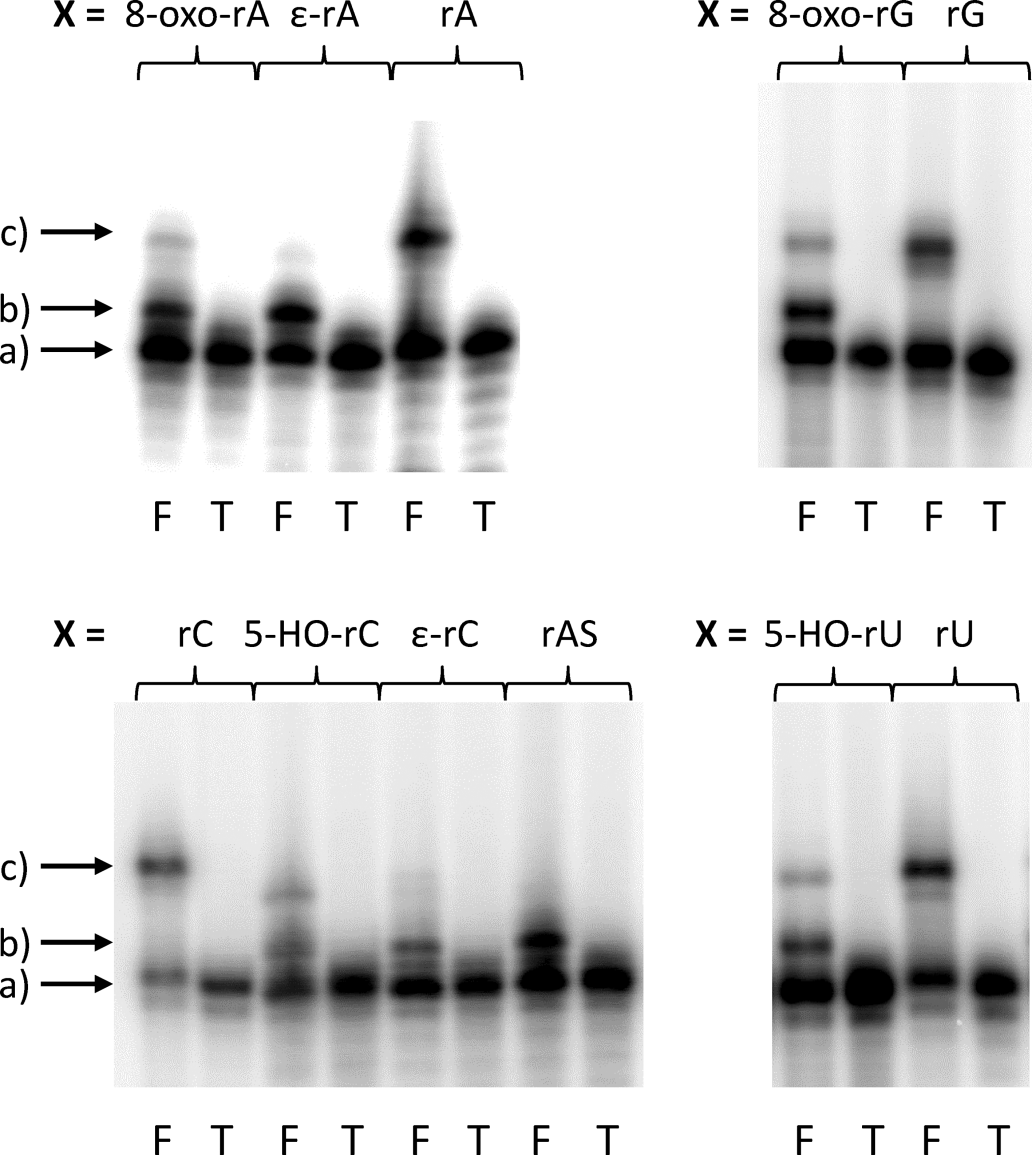
Fusion-translation products of templates bearing a natural base (rA, rC, rG, rU) or a lesion corresponding to the position X. T = mRNA construct; F = fusion translation. Arrows: a) = template; b) = truncated fusion products; and c) = full-length fusion products.

Next we decided to extend the assay to the full library of lesions depicted in Figure 2. For each lesion, the corresponding natural base was used as a control. The corresponding mRNA constructs were ^32^P-labeled and the fusion products compared to the untranslated mRNA constructs by PAGE (Figure 6). For all lesions investigated a new band with intermediate mobility between the mRNA construct and the fully translated fusion product is visible. This band can be attributed to aborted ribosomal protein synthesis at the site of the lesion. The ratio between the fusion-translation product and the intermediate band depends on the nature of the lesion.

A detailed analysis clearly shows that potentially non-coding lesions, such as rAS, ε-rA and ε-rC, do completely abort protein synthesis at the site of the lesion. Indeed, no fully translated product can be observed, and the only band that can be detected is the one corresponding to the truncated product. In all the other cases, both the fully translated and the truncated products were formed in different ratios. Interestingly, also pyrimidine lesions that show no obvious change in their coding properties or their conformational preferences around the glycosidic bond such as 5-HO-rU and 5-HO-rC show significant interference with translation.

## DISCUSSION

While there is precedence in the literature on the inhibitory action of mRNA base-lesions on translation (24,25) there is a clear lack of understanding the biochemistry behind these effects in sufficient detail. Herein we report on a versatile method to study the translation of mRNA containing classical base-lesions on a molecular level. This method is based on the well-known mRNA display technology and is characterized by covalently linking a translated peptide to the corresponding synthetic, mRNA containing the base-lesions in predefined positions. The reduced size of such mRNA constructs allows the use of gel electrophoresis combined with different labeling strategies to evaluate the outcome of translation. Theoretically this method should also allow to identify the exact composition of the attached peptide if mass spectrometric tools were used. This, however, was unsuccessful so far in our hands, most likely due to the structural complexity of the peptide–oligonucleotide conjugates.

We find that all the lesions tested show an important effect on the translation process (Figure 6). In all cases, an intermediate band between the template and the full fusion-translation process appears. Quite interestingly, the same effect is observed also with 5-HO-rU, although it is known that this lesion does not have any effect on its coding properties as evidenced from the duplex stability studies (20,33). The 5-OH group is quite far away from the hydrogen bonding site and the hydroxyl group does not interfere with them, nor does it lead to alternative tautomeric forms (34,35). In comparison with a template containing a natural rU, 5-HO-rU shows decreased translation efficiency and formation of a new band in between the template and the full-length translation band. This species forms the most intense band and can be explained by stalling ribosomal translation upon encountering the lesion. The fact that this effect is observed with 5-HO-rU demonstrates that ribosomal recognition of the nucleobases is extremely sensitive not only to the coding edge of the bases.

The investigated base lesions can be classified in two groups, those that give after translation a mixture of full-length and truncated product and those leading to a truncated translation product only. In particular ε-rA, ε-rC and abasic site rAS are belonging to the second class, causing 100% abortion of protein synthesis at the site of lesion. All of them are non-coding lesions since they have no ability to natural base recognition. They are also known to have the biggest effects on duplex stability and in reverse transcription. The other lesions that maintain the original hydrogen bonding patterns slow down the translation process, but do not interrupt it completely. Thus, their impact is less severe compared to that of the non-coding lesions.

An interesting feature was observed for the 5-HO-rC (Figure 6). When deprotected 5-HO-rC is used it shows a behavior similar to the other lesion tested here. On the other hand, when the translation was carried out using the 5-O-protected base, an efficiency similar to that of natural rC could be observed. This points to the fact that the 5-OH group interacts in the ribosome binding site, most likely via H-bonding interfering with the readout of the coding edge of the base.

## CONCLUSIONS

We have developed a method that can be used to investigate the effect of RNA lesions on ribosomal translation. So far, this has only rarely been investigated in the context of randomly oxidized mRNAs (24,25) but never at high resolution with defined base lesions in preselected positions of a synthetic mRNA construct. The results obtained here show that the investigated, commonly known base lesions can be divided into two groups, the non-coding lesions (etheno lesions and abasic site) that interrupt the translation process quantitatively, and other, less severe lesions that lead to a mixture of full-length and truncated peptides. The results obtained for 5-HO-rU and 5-HO-rC show that even remote base modifications that do not interfere with the base recognition edge lead to inefficient translation.

With the reported synthetic mRNA constructs it is in principle possible to investigate the effect of base-lesions in any position of the triplet codon. In addition, the fidelity of *trans*-lesion peptide synthesis could be determined in combination with mass spectrometric analysis of the translation products. With this we aim at understanding the biological consequences of base-damaged mRNA on translation at a molecular level.

## SUPPLEMENTARY DATA

Supplementary Data are available at NAR Online.

SUPPLEMENTARY DATA
