# Supplementary material for: The effect of RNA base lesions on mRNA translation
Source: Nucleic Acids Res. 2015 Apr 20;43(9):4713–20. doi: 10.1093/nar/gkv377 (PMC4482091; doi:10.1093/nar/gkv377)
Supplement: SUPPLEMENTARY DATA [file supp_43_9_4713__index.html]

The effect of RNA base lesions on mRNA translation — The effect of RNA base lesions on mRNA translation — SUPPLEMENTARY DATA 

# The effect of RNA base lesions on mRNA translation

## SUPPLEMENTARY DATA

**Files in this Data Supplement:**

- SUPPLEMENTARY DATA
