# Supplementary material for: The effect of RNA base lesions on mRNA translation
Source: Nucleic Acids Res. 2015 Apr 20;43(9):4713–20. doi: 10.1093/nar/gkv377 (PMC4482091; doi:10.1093/nar/gkv377)
Supplement: SUPPLEMENTARY DATA [file supp_gkv377_nar-00750-r-2015-File003.pdf]

Supplementary Information

Synthesis of the 8-oxo-rG phosphoramidite building block

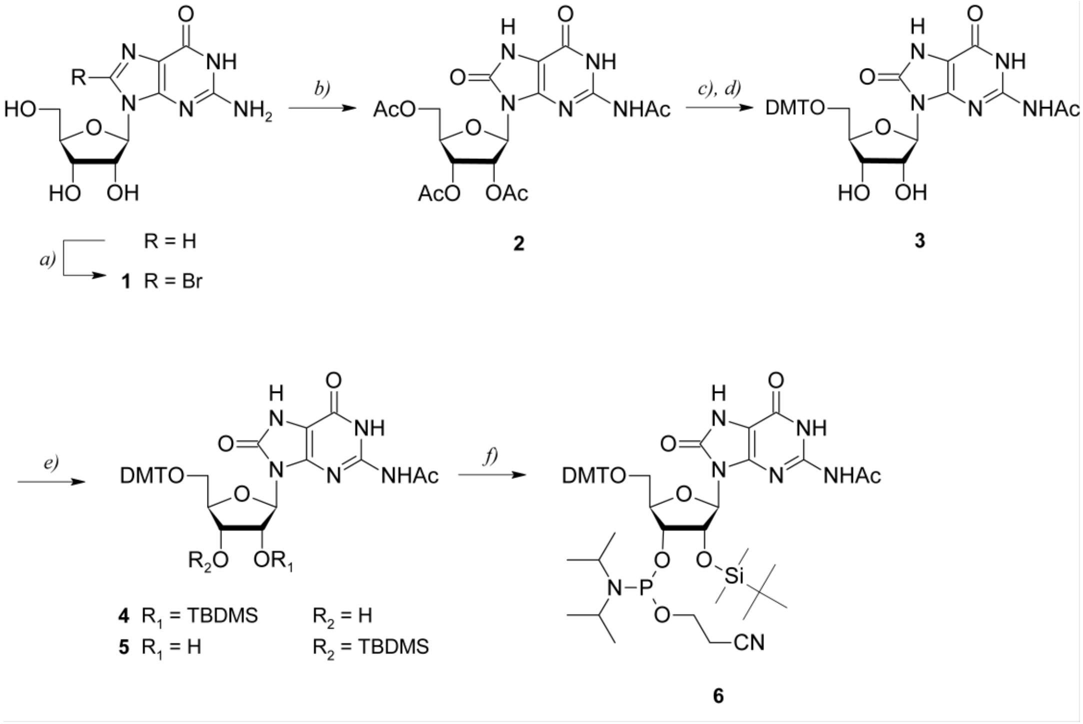

Figure S 1: a) Br_2-water, H_2O, rt; b) Ac_2O, AcOH, NaOAc, 4 h, reflux; c) KOtBu, MeOH, 15 min, rt; d); e) 4,4'-dimethoxytriphenyl chloride, pyridine, rt, 45 min; f) tert-butyldimethylsilyl chloride, $AgNO_3$, pyridine, THF, rt, 5.5 h; g) 2-cyanoethyl diisopropylchlorophosphoramidite, 1-methylimidazole, sym-collidine, THF, rt, 2 h. DMT = 4,4'-dimethoxytriphenyl, Ac = acetyl, TBDMS = tert-butyldimethylsilyl.

8-Bromoguanosine (1)

This compound was synthesized after a published method.(1)

^{1}H-NMR (300 MHz, DMSO-d_6): δ 10.81 (s, 1H, H-N(1)), 6.48 (s, 2H, H_2-N(3)), 5.68 (d, *J* = 6.34 Hz, 1H, H-C(1')), 5.43 (d, *J* = 6.20 Hz, 1H), 5.07 (d, *J* = 5.02 Hz, 1H), 5.00 (dd, *J* = 11.88, 6.03 Hz, 1H), 4.90 (dd, *J* = 6.42, 5.58 Hz, 1H), 4.13 (dd, *J* = 8.48, 4.97 Hz, 1H), 3.87-3.82 (m, 1H), 3.68-3.61 (m, 1H), 3.54-3.46 (m, 1H).

^{13}C-NMR (75 MHz, DMSO-d_6): δ 155.5, 153.6, 152.2, 121.2, 117.6, 89.8, 86.0, 70.6, 70.4, 62.1.

7-Hydro-2-*N*,2'-*O*,3'-*O*,5'-*O*-tetraacetylguanosine-8-one (2)

This synthesis of this compound was adapted from a published method.(2)

To a mixture of 8-bromoguanosine **1** (5 g, 14 mmol) and NaOAc (20.7 g, 250 mmol, 18 eq), acetic anhydride (175 ml) and acetic acid (175 ml) were added. The resulting solution (above ~ 80 °C) was then heated for 4 hours under reflux (140 °C bath temperature) and cooled to room temperature. The colorless solution was evaporated under reduced pressure and residual solvents were removed by coevaporation with ethanol (2 x 50 ml). The colorless residue was dispersed in $CHCl_3$ (200 ml) and water (100 ml) and the organic phase was washed with sat. $NaHCO_3$ (2 x 100 ml) and water (100 ml). The organic phase was dried over Na_2SO_4, filtered and the solvents were evaporated. The pale yellow foam was finally chromatographed on silica gel (75 g, eluted with a gradient from CH_2Cl_2 to CH_2Cl_2/MeOH 9:1) to give **2** (5.86 g, 91 %) as a colorless foam.

TLC (CH_2Cl_2/MeOH 9:1): R_f = 0.55

^{1}H-NMR (300 MHz, $CDCl_3$): δ 12.05 (1H, H-N(2)), 9.94 (1H, H-N(7)), 9.30 (1H, H-N(1)), 6.10 (1H, H-C(1')), 5.93-5.90 (1H, H-C(2')), 5.71-5.70 (1H, H-C(3')), 4.70-4.62 (1H, H-C(4')), 4.42-4.35 (2H, H_2-C(5')), 2.29 (s, 3H, Ac), 2.11-2.06 (m, 12H, 3 x Ac).

^{13}C-NMR (75 MHz, $CDCl_3$): δ 172.5, 171.5, 169.8, 169.7 (4 x C=O, Ac), 151.9 (C-6), 150.0 (C-2), 146.8 (C-4), 144.5 (C-8), 104.7 (C-5), 83.9 (C-1'), 78.9 (C-4'), 71.7 (C-3'), 70.9 (C-2'), 63.4 (C-5'), 24.0 (CH_3, NHAc), 20.8, 20.4, 20.3 (3 x CH_3, OAc).

5′-*O*-(4,4′-Dimethoxytriphenylmethyl)-7-hydro-2-*N*-acetylguanosine-8-one (3)

Amberlite IR-120 in pyridinium form was prepared as follows: the resin was put in a column (diameter 2 cm, length 10 cm) and washed with pyridine:water = 1:1 (400 ml) followed by water, until neutral pH was achieved. The resin obtained was stored and used later.(3)

Compound **2** (4.5 g, 9.6 mmol) was added to a freshly prepared solution of KOtBu (1.4 g, 12.48) in methanol (140 ml). Soon a colorless precipitate formed. After 10 minutes, more methanol (140 ml) was added and the precipitate dissolved again after short sonication. The reaction was complete according to TLC after another 10 minutes and the reaction was quenched by addition of the previously prepared Amberlite IR-120 in pyridinium form until pH 7. The resin was filtered off and washed with methanol. The solvents were then removed under reduced pressure and the residue was coevaporated with pyridine.

The intermediate, obtained as an oil, was dissolved in dry pyridine (52 ml), and 4,4'-dimethoxytriphenyl chloride (3 g, 8.8 mmol) was added to the solution and stirred at room temperature for 30 min. A second batch of 4,4'-dimethoxytriphenyl chloride (3 g, 8.8 mmol) was added, and the reaction mixture was stirred for another 15 min. When the reaction was complete, 15 ml of ethanol were added and solvents were directly evaporated. The residual oil was dissolved in methylene chloride and extracted with sat. $NaHCO_3$ (2 times) and sat. NaCl (2 times). The organic phase was dried over Na_2SO_4, filtered and evaporated. The product was purified by flash chromatography (eluted with a gradient from CH_2Cl_2/MeOH 96:4 to 94:6 to 90:10 with 0.1% triethylamine) resulting **3** (4.17 g, 68%) as a colorless foam.

TLC (CH_2Cl_2/MeOH 9:1): R_f = 0.37

^{1}H NMR (400 MHz, DMSO-d_6) δ 12.08, 11.49, 11.25 (3s br, 3xNH), 7.36-7.34 (m, 2H, DMT), 7.23-7.15 (m, 7H, DMT), 6.82-6.76 (m, 4H, DMT), 5.66 (d, J = 3.7 Hz, 1H, H-1'), 5.37 (d, J = 4.4 Hz, 1H, HO-2'), 4.90 (d, J = 6.4 Hz, 1H, HO-3'), 4.74 (m, 1H, H-2'), 4.26 (m, 1H, H-3'), 3.93 (m, 1H, H-4'), 3.72, 3.71 (2s, 6H, OMe), 3.24 – 3.16 (m, 1H, H-5'), 3.12 (dd, J = 10.1, 3.0 Hz, 1H, H-5'), 2.14 (s, 3H, Ac).

^{13}C NMR (101 MHz, DMSO- d_6) δ 173.20 (s), 157.94 (s), 157.90 (s), 151.41 (s), 149.20 (s), 147.05 (s), 144.97 (s), 144.80 (s), 135.71 (s), 135.66 (s), 129.76 (d), 129.67 (d), 127.77 (d), 127.56 (d), 126.46 (d), 112.94 (d), 112.87 (d), 103.56 (d), 86.65 (t, C-1'), 85.22 (s, DMT), 82.48 (d, C-4'), 70.59 (d, C-2'), 70.43 (d, C-3'), 64.60 (t, C-5'), 54.94, 54.91 (2q, OMe), 23.70 (q, Ac)

NSI$^+$-HRMS calculated for $C_{33}H_{33}N_5O_9Na$ ($[M+Na]^+$): 666.2170, found: 666.2185

5′-*O*-(4,4′-Dimethoxytriphenylmethyl) 2',3'-O-tert-butyldimethylsilyl-8-oxo-guanosine (4,5)

Compound **3** (1 g, 1.56 mmol) was dissolved in dry THF (30 ml). $AgNO_3$ (0.80 g, 4.70 mmol) and pyridine (1.4 ml, 18.6 mmol) were added to the solution and stirred in the dark for 15 min. When the system became homogenous, tert-butyldimethylsilyl chloride (0.81 g, 5.34 mmol) was added, and the reaction mixture was stirred for 5.5 hours. When the reaction was completed, the reaction was quenched by filtering the mixture over celite into 5% $NaHCO_3$. This resulting suspension was extracted twice with CH_2Cl_2, and the organic phase was washed twice with sat. $NaHCO_3$ and twice with sat. NaCl, dried over Na_2SO_4, filtered and

evaporated. The product was purified by flash chromatography (eluted with a gradient from CH_2Cl_2/MeOH 99:1 to 97:3 with 0.1% triethylamine), obtaining products as white solids (**5**: 157 mg, 15 %, **4**: 767 mg, 65%).

Analytical data for compound 4

TLC (CH_2Cl_2/MeOH 9:1): R_f = 0.60

^{1}H NMR (400 MHz, DMSO- d_6) δ 12.09, 11.55, 11.29 (3s, 3xNH), 7.73-7.35 (m, 2H, DMT), 7.25-7.16 (m, 7H, DMT), 6.83-6.78 (m, 4H, DMT), 5.65 (d, J = 4.1 Hz, 1H, H-1'), 4.92 (s, 1H, H-2'), 4.72 (s, 1H, HO-3'), 4.26 (dd, J = 12.2, 5.9 Hz, 1H, H3'), 3.93 (dd, J = 10.5, 5.2 Hz, 1H, H-4'), 3.72, 3.71 (2s, 6H, OMe), 3.17 (d, J = 2.7 Hz, 2H, H-5'), 2.14 (s, 3H, Ac), 0.82 (s, 9H, TBS), 0.02, -0.03 (2s, 3H, TBS).

^{13}C NMR (101 MHz, DMSO- d_6) δ 173.28 (s), 157.95 (s), 157.92 (s), 151.18 (s), 149.02 (s), 147.07 (s), 144.94 (s), 144.85 (s), 135.65 (s), 135.61 (s), 129.75 (d), 129.69 (d), 127.76 (d), 127.59 (d), 126.49 (d), 112.97 (d), 112.92 (d), 103.52 (d), 86.40 (d, C-1'), 85.23 (s, DMT), 82.43 (d, C-4'), 72.12 (d, C-2'), 70.23 (d, C-3'), 64.00 (t, C-5'), 54.95, 54.93 (2q, OMe), 25.65 (q, TBS), 23.68 (q, Ac), -4.76, -5.07 (q, TBS).

NSI^+-HRMS calculated for $C_{39}H_{47}O_9N_5NaSi$ ($[M+Na]^+$): 780.3035, found: 780.3051

Analytical data for compound 5

TLC (CH_2Cl_2/MeOH 9:1): R_f = 0.54

^{1}H NMR (400 MHz, DMSO- d_6) δ 12.08, 11.65, 11.22 (3s br, 3xNH), 7.37-7.34 (m, 2H, DMT), 7.26-7.16 (m, 7H, DMT), 6.82-6.79 (m, 4H, DMT), 5.60 (d, J = 4.6 Hz, 1H, H-1'), 5.18 (d, J = 6.1 Hz, 1H, HO-2'), 4.98 (dd, J = 10.4, 5.2 Hz, 1H, H-2'), 4.36 (t, J = 5.0 Hz, 1H, H-3'), 3.87 (dd, J = 9.9, 5.0 Hz, 1H, H-4'), 3.71 (2s, 6H, OMe), 3.23 (dd, J = 10.4, 4.2 Hz, 1H, H-5'), 3.03 (dd, J = 10.2, 5.8 Hz, 1H, H-5'), 2.16 (s, 3H, Ac), 0.80 (s, 9H, TBS), 0.03, -0.02 (2s, 6H, TBS).

^{13}C NMR (101 MHz, DMSO) δ 173.18 (s), 157.99 (s), 157.97 (s), 151.16 (s), 149.20 (s), 147.21 (s), 145.18 (s), 144.86 (s), 135.69 (s), 135.58 (s), 129.66 (d), 129.59 (d), 127.70 (d), 127.62 (d), 126.55 (d), 113.00 (d), 112.97 (d), 103.45 (d), 86.20 (d, C-1'), 85.44 (s, DMT), 82.47 (d, C-4'), 72.38 (d, C-3'), 69.27 (d, C-2'), 63.67 (t, C-5'), 54.95 (q, OMe), 25.73 (q, TBS), 23.70 (q, Ac), -4.54, -5.20 (q, TBS).

5′-*O*-(4,4′-Dimethoxytriphenyl) 2'-O-tert-butyldimethylsilyl-3'-O-(diisopropylamino cyanoethoxy)-phosphino-8-oxo-guanine (6)

To a solution of compound **4** (500 mg, 0.66 mmol) in dry THF (10 ml), sym-collidine (654 µl, 4.95 mmol) and 1-methylimidazole (26 µl, 0.33 mmol) were added. Once everything solubilized, 2-cyanoethyl diisopropylamino chlorophosphine (368 µl, 1.65 mmol) was slowly added over 5 minutes. A white precipitate formed during the reaction and after 2 hours, the reaction was quenched with ethanol (2 ml) at 0 °C. The suspension was taken up in CH_2Cl_2 and washed twice with sat. $NaHCO_3$ and sat. NaCl. The organic phase was dried over Na_2SO_4, filtered and evaporated to dryness. The residue was purified by flash chromatography (eluted with ethyl acetate: hexane = 1:1), and since this was not sufficient, a second purification had to be done (eluted with a gradient from CH_2Cl_2 to CH_2Cl_2/MeOH 97:3). Finally, the product was dissolved in ethyl acetate and precipitated from cold hexane. Phosphoramidite **6** was obtained as a fine colorless solid (400 mg, 64%).

TLC (CH_2Cl_2/MeOH 19:1): R_f = 0.38

^{1}H NMR (300 MHz, $CDCl_3$) δ 7.67 – 7.01 (m, 9H + residual $CHCl_3$), 6.84 – 6.63 (m, 4H), 5.90 – 5.73 (m, 1H), 5.33 – 5.13 (m, 1H), 4.42 – 3.80 (m, 4H), 3.70 (s, 3H), 3.68 (s, 3H), 3.60 – 3.32 (m, 4H), 3.15 – 2.84 (m, 1H), 2.73-2.61 (m, 1H), 1.20 – 1 (m, 12H), 0.9-0.2 (m, 9H), 0.1-0.2 (m, 6H).

^{13}C NMR (101 MHz, $CDCl_3$) δ 171.68, 171.44, 158.92, 158.89, 158.81, 152.24, 151.84, 149.96, 146.41, 146.02, 145.89, 145.70, 145.50, 145.35, 136.80, 136.40, 136.23, 135.97, 130.27, 130.23, 130.18, 128.21,

128.13, 127.28, 127.17, 118.33, 117.47, 113.38, 104.79, 104.42, 86.22, 85.89, 85.62, 84.83, 83.77, 73.26, 72.80, 71.53, 70.60, 63.89, 63.47, 59.16, 59.05, 57.37, 57.17, 55.48, 55.42, 43.65, 43.52, 42.95, 42.82, 31.72, 25.99, 25.90, 25.88, 25.84, 24.88, 24.81, 24.74, 23.22, 22.97, 20.48, 20.13, 20.06, 18.19, 18.11, 14.25, -4.28, -4.74, -4.85.

^{31}P NMR (122 MHz, $CDCl_3$) δ 150.18, 148.36

NSI$^+$-HRMS calculated for $C_{48}H_{65}N_7O_{10}PSi$ ([M+H]$^+$): 958.4294, found: 958.4323

NMR Spectra of Compounds 3-6

Spectra of compound 3

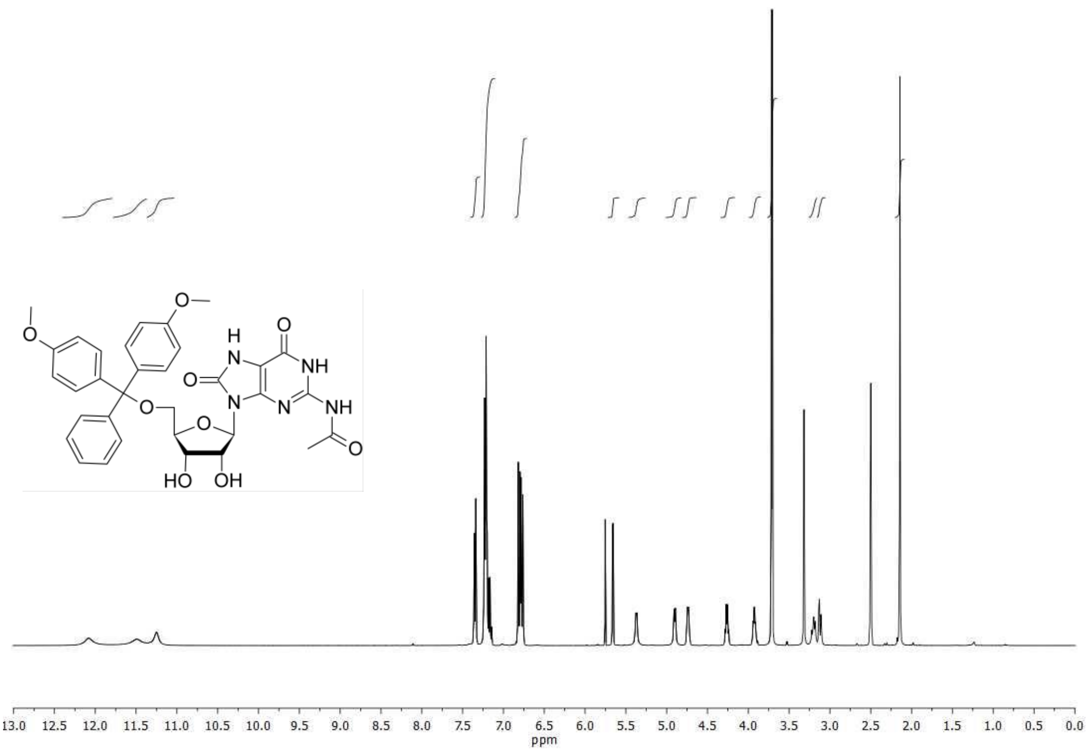

Figure S 2: ^{1}H-NMR of compound **3**

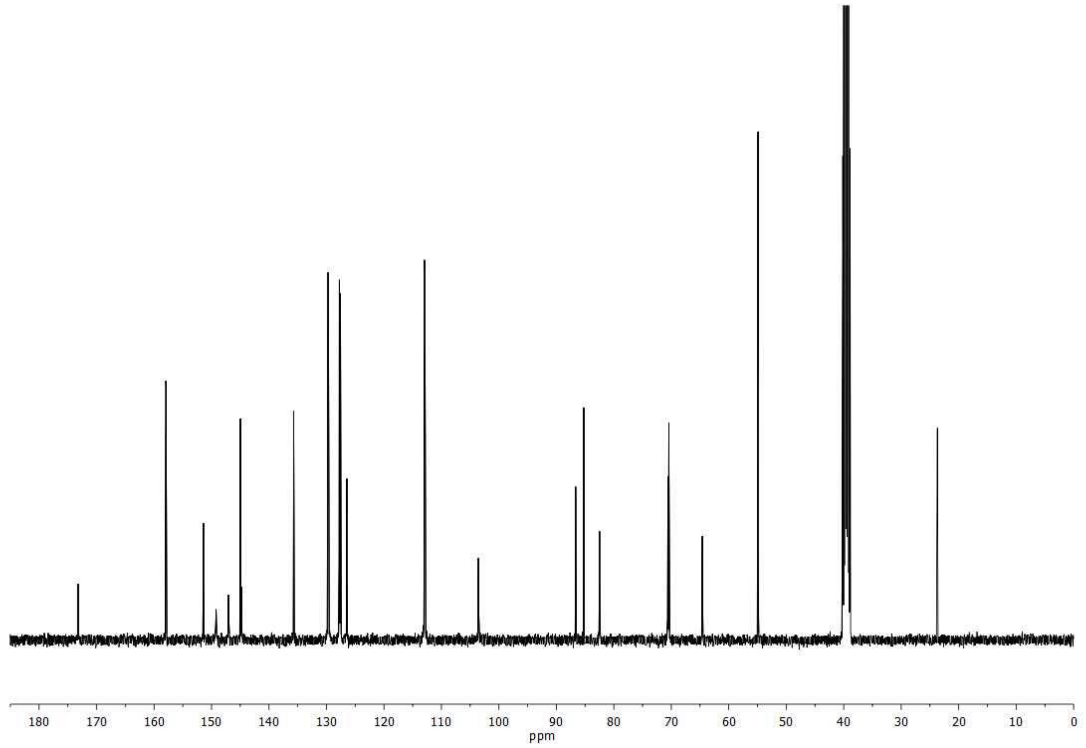

Figure S 3: ^{13}C-NMR of compound **3**

Spectra of compound 4

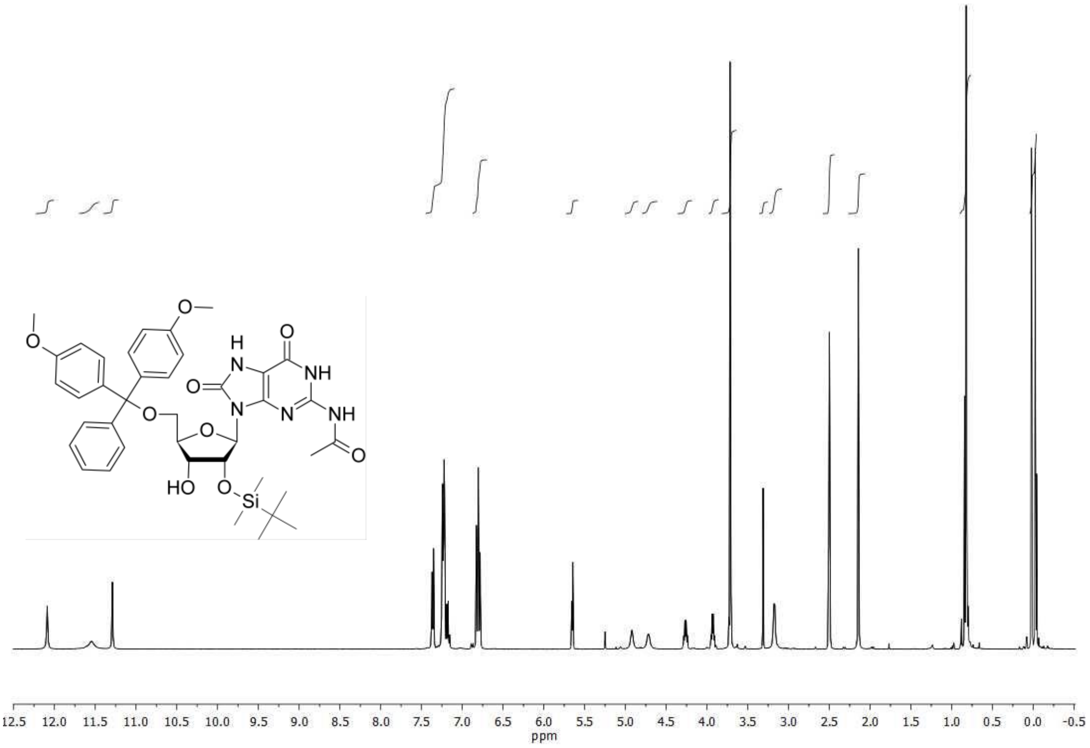

Figure S 4: ^{1}H-NMR of compound **4**

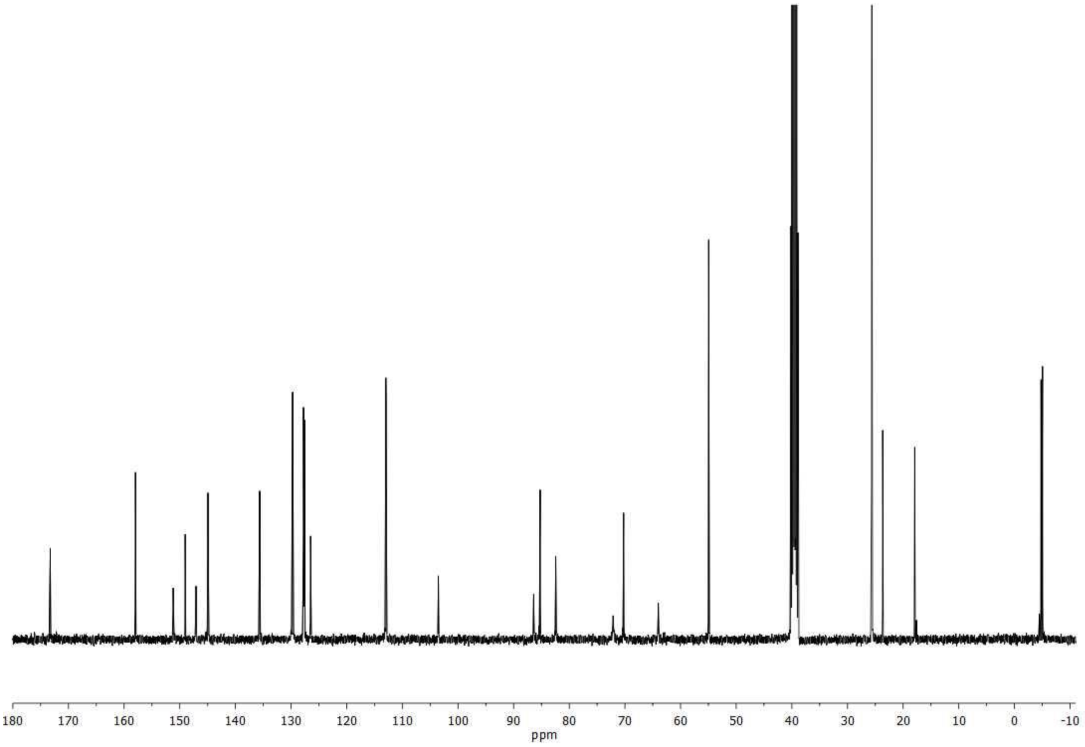

Figure S 5: ^{13}C-NMR of compound **4**

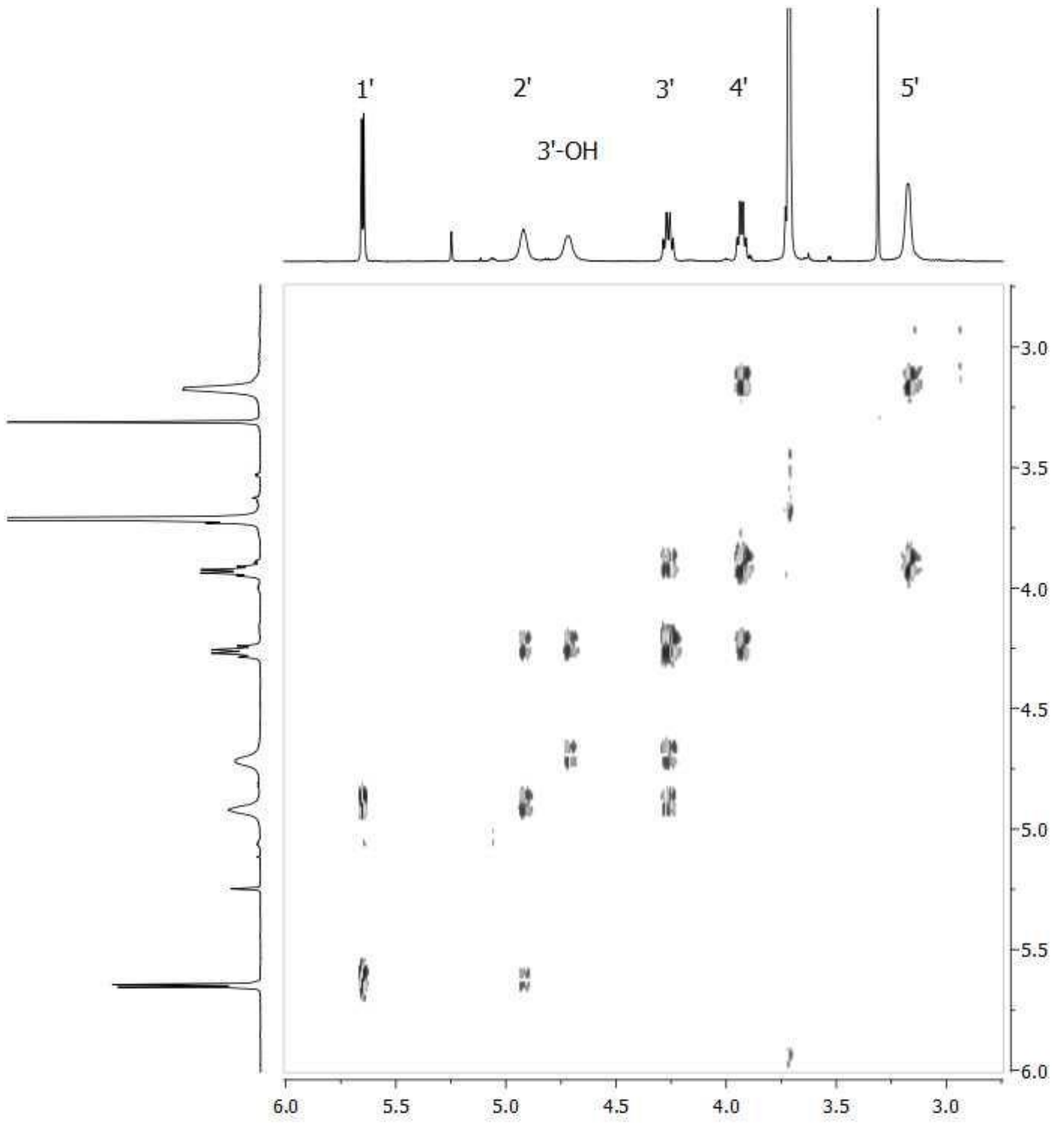

Figure S 6: $^{1}H,^{1}H$-COSY of compound **4**

Spectra of compound 5

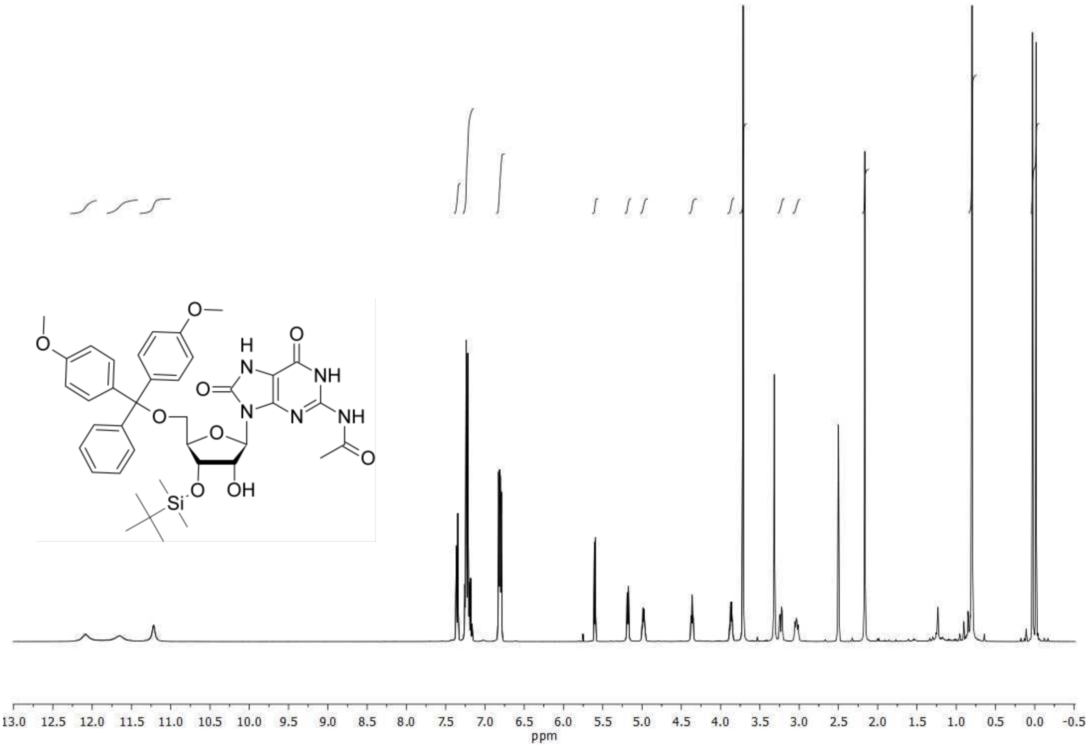

Figure S 7: ^{1}H-NMR of compound **5**

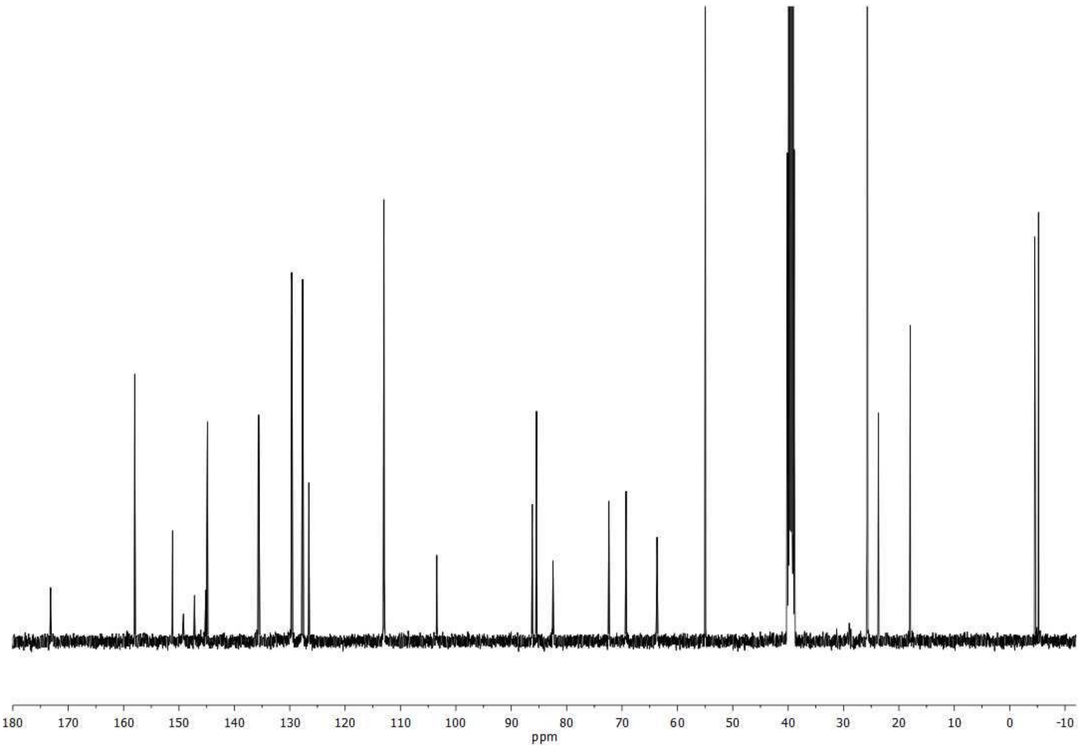

Figure S 8: ^{13}C-NMR of compound **5**

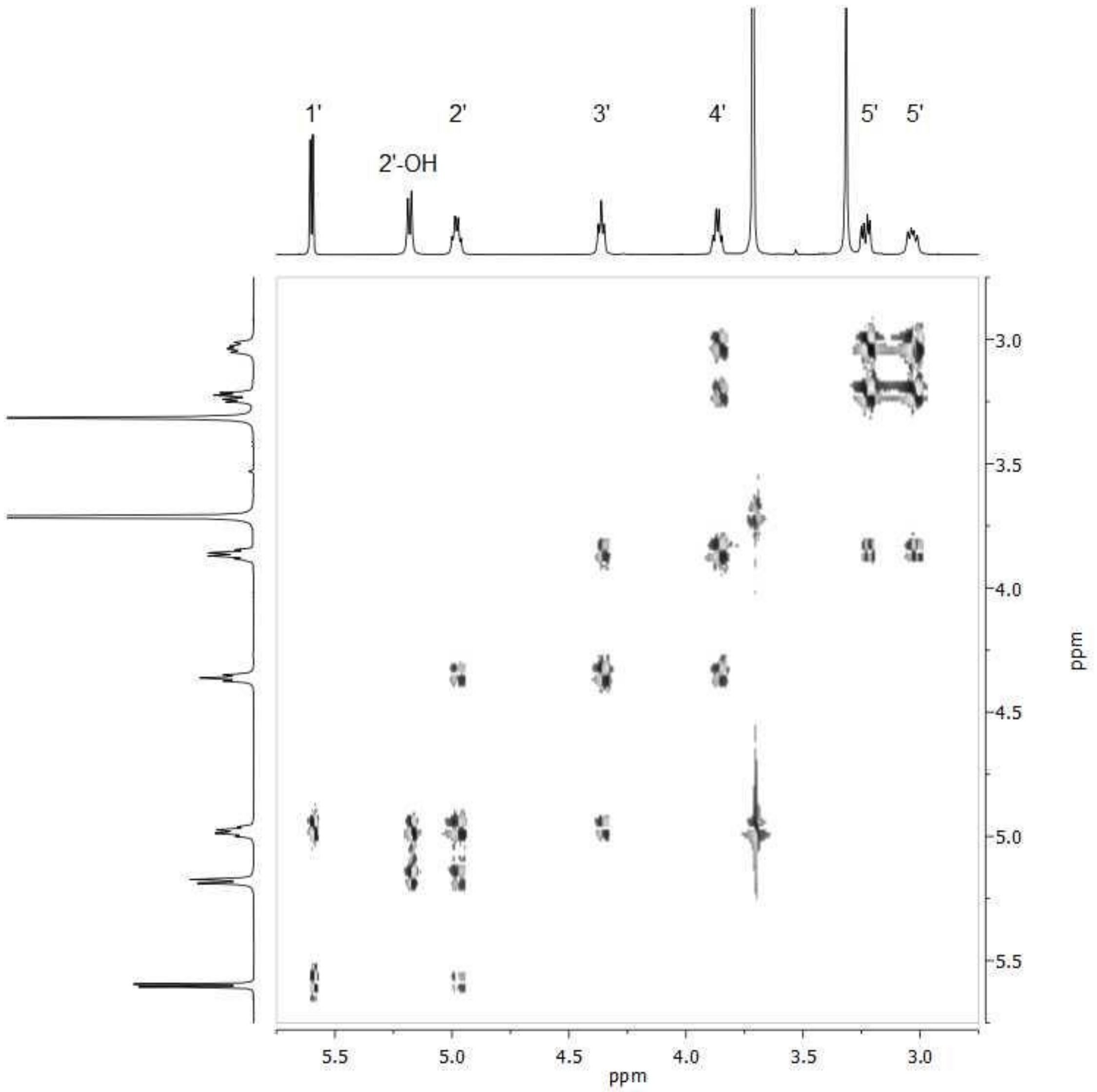

Figure S 9: ^{1}H,^{1}H-COSY of compound **5**

Spectra of compound 6

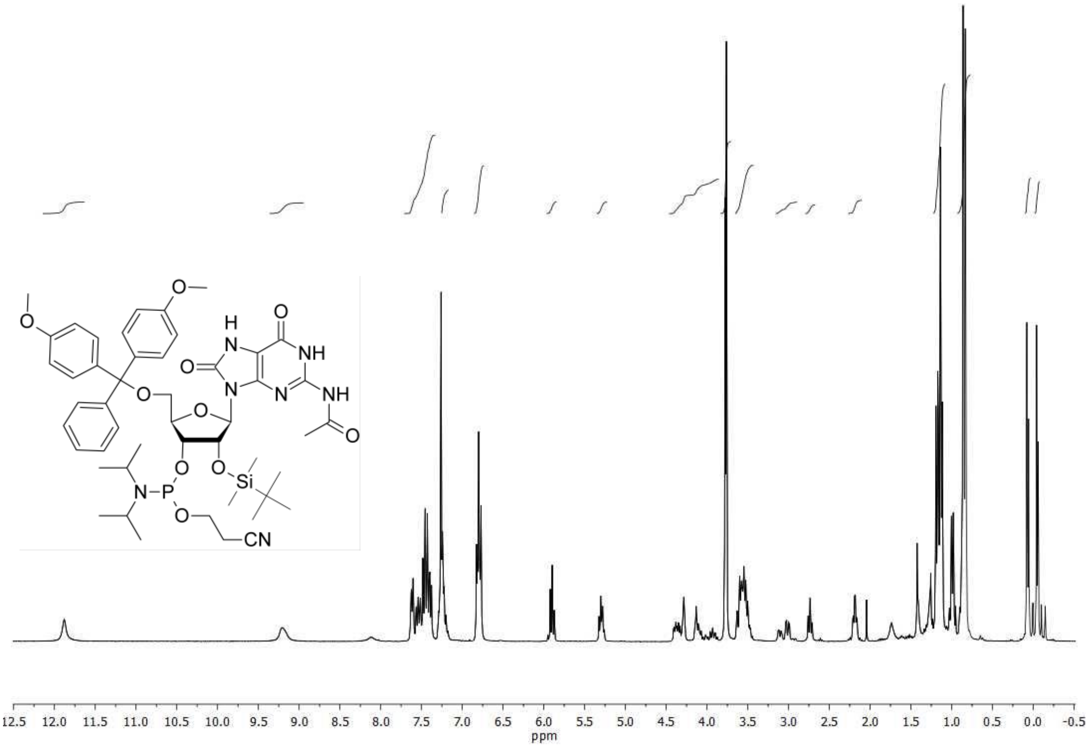

Figure S 10: ^{1}H-NMR of compound **6**

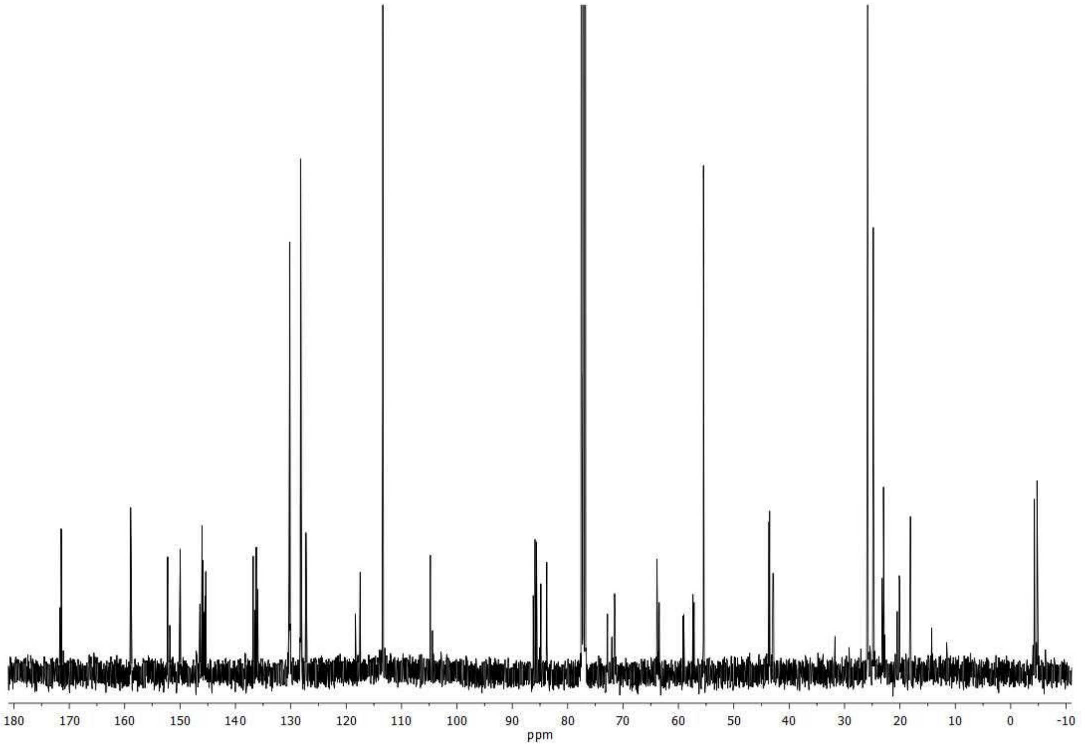

Figure S 11: ^{13}C-NMR of compound **6**

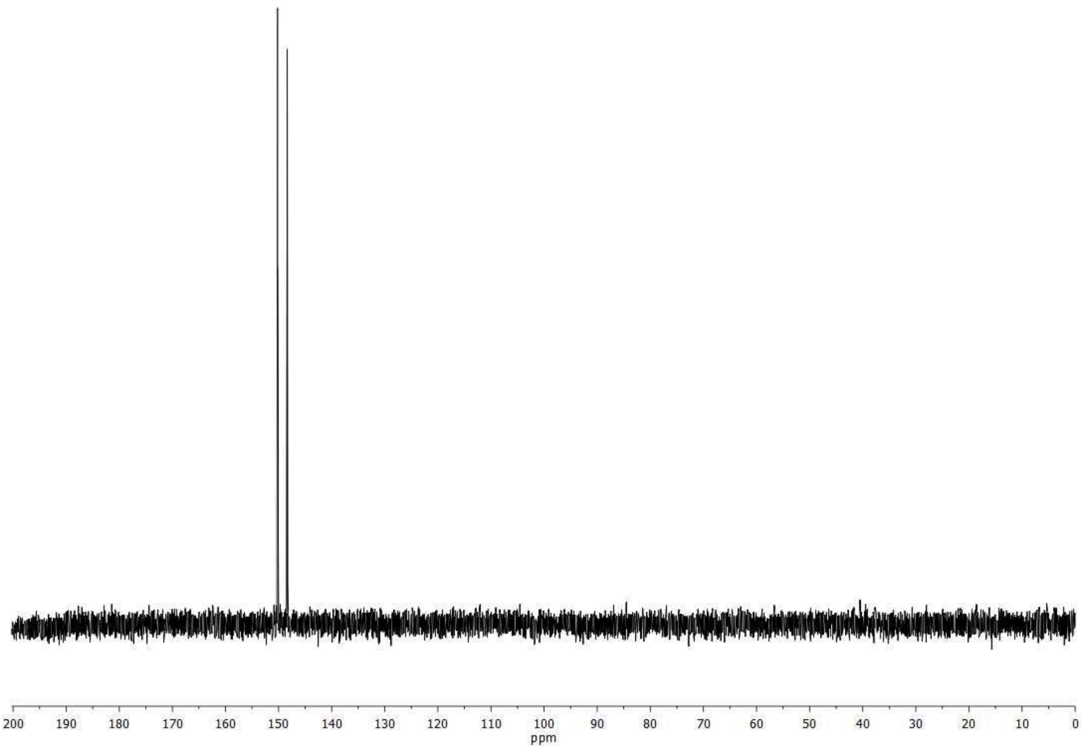

Figure S 12: ^{31}P-NMR of compound **6**

Mass Spectrometry

Mass Spectrometry

Table S 1: Sequences of synthesized oligonucleotides

**linker**	**d(AAA AAA AAA AAA AAA AAA AAA) 999 dAdCdC P**
**RNA 5**	GGG AGG ACG AAA UGG AAC AGA **(ε-rA)**AC UGA UC
**RNA 6**	GGG AGG ACG AAA UGG AAC AGA **(5-HO-rU)**AC UGA UC
**RNA 7**	GGG AGG ACG AAA UGG AAC AGA **(8-oxo-rG)**AC UGA UC
**RNA 8**	GGG AGG ACG AAA UGG AAC AGA **(5-HO-rC)**AC UGA UC
**RNA 9**	GGG AGG ACG AAA UGG AAC AGA **(8-oxo-rA)**AC UGA UC
**RNA 10**	GGG AGG ACG AAA UGG AAC AGA **(ε-rC)**AC UGA UC
**RNA 11**	GGG AGG ACG AAA UGG AAC AGA **(rAS)**AC UGA UC
P = puromycin, 9 = triethylene glycol spacer	

Linker (Calculated mass = 8576.97 Da). Calculated m/z for $[M-17H]^{17-}$ 503.5; $[M-16H]^{16-}$ 535.1; $[M-15H]^{15-}$ 570.8; $[M-14H]^{14-}$ 611.6; $[M-13H]^{13-}$ 658.8; $[M-12H]^{12-}$ 713.7; $[M-11H]^{11-}$ 778.7; $[M-10H]^{10-}$ 856.7; $[M-9H]^{9-}$ 952.0. Found: 503.6; 535.1; 570.8; 611.6; 658.6; 778.7; 856.7; 952.0.

RNA 5 (Calculated mass = 9503.94 Da). Calculated m/z for $[M-15H]^{15-}$ 632.5; $[M-14H]^{14-}$ 677.9; $[M-13H]^{13-}$ 730.1; $[M-12H]^{12-}$ 791.0; $[M-11H]^{11-}$ 863.0. Found: 632.6; 677.9; 730.0; 791.0; 862.9.

RNA 6. **(Calculated mass = 9472.88 Da)**. Calculated m/z for $[M+Na-15H]^{15-}$ 630.5; $[M-14H]^{14-}$ 675.6; $[M-13H]^{13-}$ 727.7 $[M-12H]^{12-}$ 788.4; $[M+Na-10H]^{10-}$ 946.3. Found: 630.5; 675.6; 727.7; 788.3; 946.2.

RNA 7 (Calculated mass = 9511.92 Da). Calculated m/z for; $[M-7H]^{7-}$ 1357.85; $[M-6H]^{6-}$ 1901.384; $[M-4H]^{4-}$ 2376.98. Found: 1357.80; 1901.30; 2376.88

RNA 8 (Calculated mass = 9067.0 Da). Calculated m/z for $[M+Na-16H]^{16-}$ 600.8; $[M+2Na-15H]^{15-}$ 642.4; $[M+2Na-14H]^{14-}$ 688.3; $[M+2Na-13H]^{13-}$ 741.4; $[M+Na-12H]^{12-}$ 801.4. Found: 600.8; 642.4; 688.2; 741.4 801.3.

RNA 9 (Calculated mass = 9495.42 Da). Calculated m/z for $[M+2Na-16H]^{16-}$ 595.2; $[M+Na-15H]^{15-}$ 633.5; $[M+2Na-15H]^{15-}$ 634.9; $[M-14H]^{14-}$ 677.3; $[M+Na-13H]^{13-}$ 731.1; $^{-}$. Found: 595.3; 633.4; 635.0, 677.3; 731.1

RNA 10 (Calculated mass = 9479.92 Da). Calculated m/z for $[M+Na-16H]^{16-}$ 592.9; $[M+Na-15H]^{15-}$ 632.4; $[M+Na-14H]^{14-}$ 677.7; $[M+Na-13H]^{13-}$ 729.9; $[M+Na-12H]^{12-}$ 790.8; $[M+Na-11H]^{11-}$. Found: 593.0; 632.3; 677.7; 729.8; 790.7.

RNA11 (Calculated mass = 9511.96 Da). Calculated m/z for $[M-16H]^{16-}$ 593.5; $[M-15H]^{15-}$ 633.1; $[M-14H]^{14-}$ 678.4; $[M-13H]^{13-}$ 730.7; $[M-12H]^{12-}$ 791.7;. Found: 593.5; 633.2; 678.5; 730.6; 791.3.

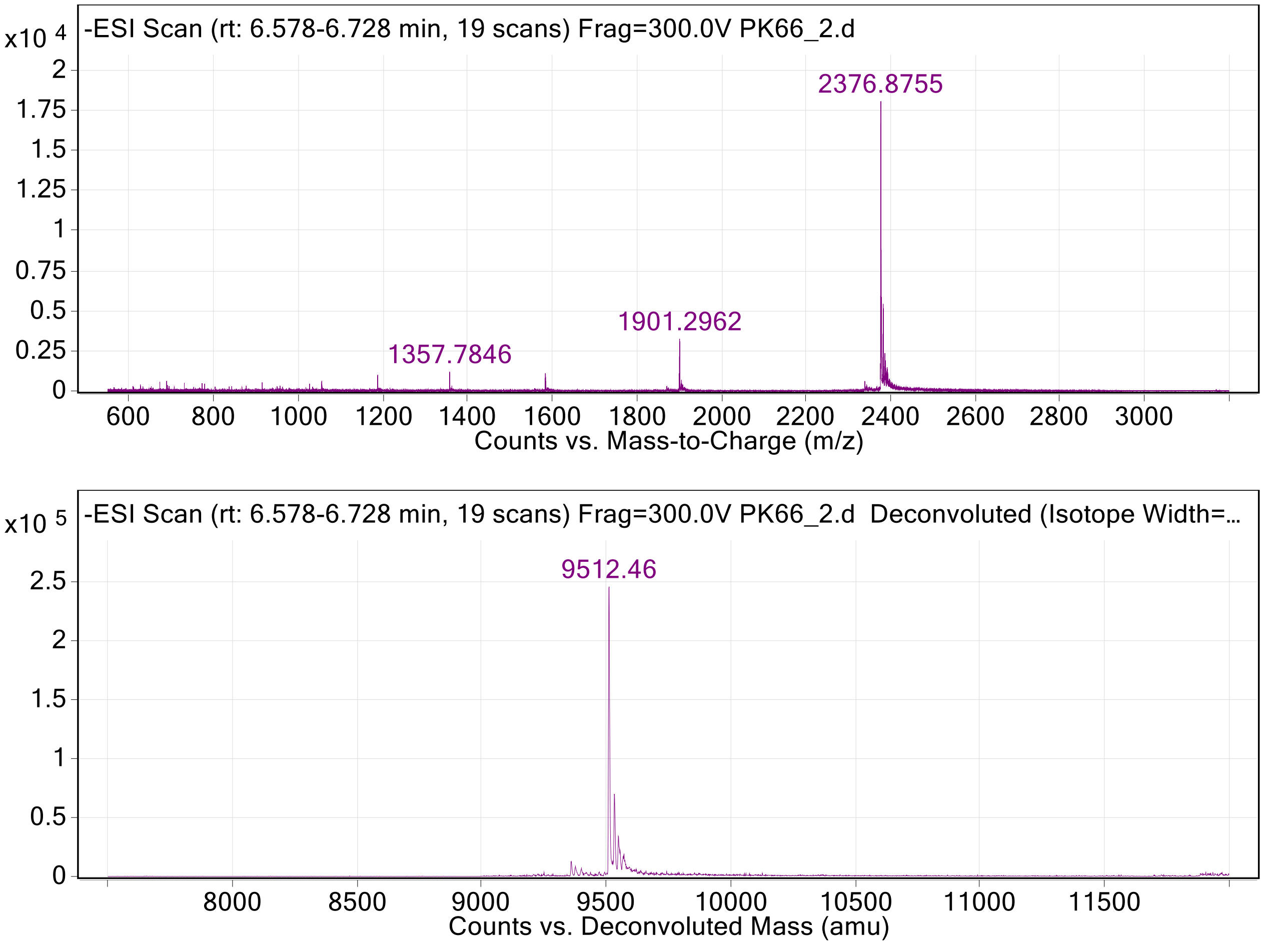

Figure S 13: ESI$^-$-MS spectrum (upper) and the deconvolution (lower) for RNA 7

Splint ligation

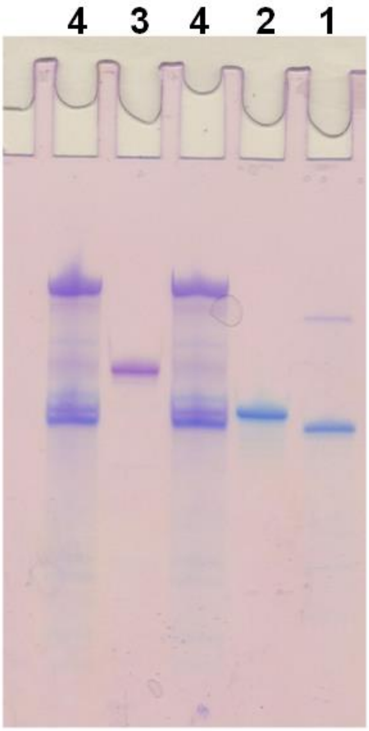

Figure S 14: PAGE minigel of splint ligation products and components. Lane 1: splint; lane 2: DNA linker; lane 3: RNA oligonucleotide; lanes 4: splint-ligation product. The gel is stained with Stains-All solution (Sigma-Aldrich).

Gel Quantification

Quantification of fusion-translation assays

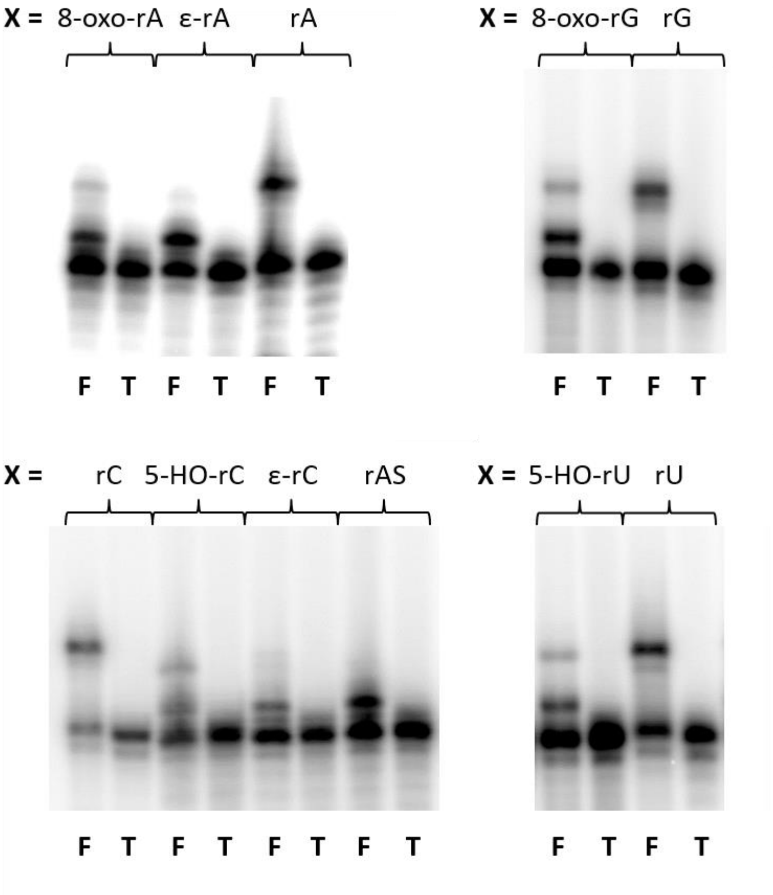

Figure S 15: Fusion-translation products of templates bearing a natural base or a lesion in position X. T = template, F = fusion translation

Table S 2: Quantitative data of fusion translation experiments shown in Figure S 15 (normalized, in %)

X=	Untranslated template	Truncated translation	Full translation
**rA**	68	0	32
**8-oxo-rA**	62	32	5
**ε-rA**	54	46	0
**rC**	45	0	55
**5-HO-rC**	57	31	11
**ε-rC**	71	29	0
**rAS**	58	42	0
**rG**	68	0	32
**8-oxo-rG**	57	37	6
**rU**	61	0	39
**5-HO-rU**	66	28	6

Quantification of fusion-translations with 5-HO-rC containing templates

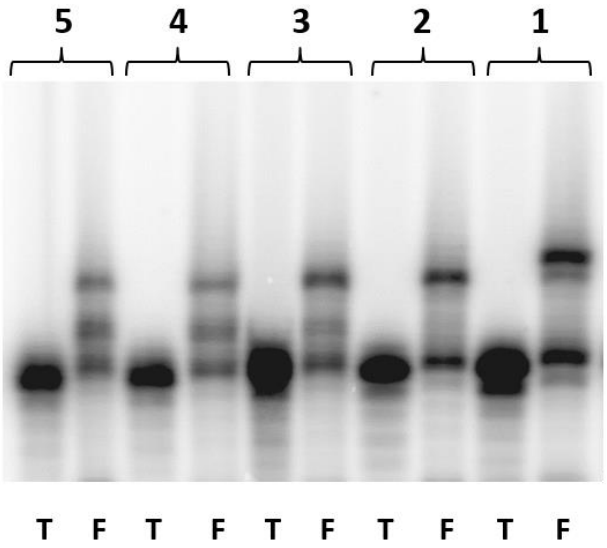

Figure S 16: Fusion-translation product obtained Fusion-translation product obtained from a template containing rC (1) or a 5-HO-rC (2-5) at the position = X. 5-HO-rC was used as protected (2), deprotected just before the translation (3), deprotected before splint ligation and warmed up to 80°C for 10 min before translation (4), and deprotected before the splint ligation (5).

Table S 3: Quantitative data of fusion-translation experiments shown in Figure S 16 (normalized, in %)

**Line**	**Untranslated template**	**Truncated translation**	**Full translation**
**1**	53	0	47
**2**	55	0	45
**3**	41	24	35
**4**	42	34	24
**5**	43	35	23

Literature

1. Long, R.A., Robins, R.K. and Townsend, L.B. (1968) In Zorbach, W. W. and Tipson, R. S. (eds.), *Synthetic Procedures in Nucleic Acid Chemistry, Vol. 1: Preparation of Purines, Pyrimidines, Nucleosides, and Nucleotides*, pp. 228-229.
2. Ikehara, M. and Maruyama, T. (1975) Studies of nucleosides and nucleotides—LXV Purine cyclonucleosides-26 a versatile method for the synthesis of purine O-cyclo-nucleosides. The first synthesis of 8, 2'-anhydro-8-oxy 9-ß-d-arabinofuranosylguanine. *Tetrahedron*, **31**, 1369-1372.
3. Roelen, H.C., Saris, C.P., Brugghe, H.F., van den Elst, H., Westra, J.G., van der Marel, G.A. and van Boom, J.H. (1991) Solid-phase synthesis of DNA fragments containing the modified base 7-hydro-8-oxo-2'-deoxyguanosine. *Nucleic Acids Res.*, **19**, 4361-4369.
